# Analysis of Phytonutrients, Anti-Mutagenic and Chemopreventive Effects of Tropical Fruit Extracts

**DOI:** 10.3390/foods10112600

**Published:** 2021-10-27

**Authors:** Piya Temviriyanukul, Suwapat Kittibunchakul, Piyapat Trisonthi, Woorawee Inthachat, Dalad Siriwan, Uthaiwan Suttisansanee

**Affiliations:** 1 Food and Nutrition Academic and Research Cluster, Institute of Nutrition, Mahidol University, Salaya, Phuttamonthon, Nakhon Pathom 73170, Thailand; piya.tem@mahidol.ac.th (P.T.); suwapat.kit@mahidol.ac.th (S.K.); woorawee.int@mahidol.ac.th (W.I.); 2 Institute of Food Research and Product Development, Kasetsart University, Chatuchak, Bangkok 10900, Thailand; piyapat.tr@ku.th

**Keywords:** antioxidant, chemoprevention, fruits, *Mangifera indica*, mutagenicity, phytochemicals, Thailand

## Abstract

Thailand is located in the tropics and a wide variety of fruits are grown commercially. However, studies regarding the phytonutrients, anti-mutagenic and chemopreventive effects of these fruits are limited. Thus, phytochemical profiles and inhibition of key enzymes involved in obesity and diabetes, together with anti-mutagenic and chemopreventive properties of eight tropical fruit extracts cultivated in Thailand, including *Psidium guajava* ‘Kimju’, *Psidium guajava* ‘Keenok’, *Ananas comosus* ‘Pattavia’, *Ananas comosus* ‘Phulae’, *Durio zibethinus* ‘Chanee’, *Durio zibethinus* ‘Monthong’, *Carica papaya* ‘Khaekdum’ and *Mangifera indica* ‘Namdokmai’ were investigated. Different cultivars were also compared. Results showed that *M. indica* ‘Namdokmai’ was the most antioxidant-rich extract containing abundant 4-hydroxybenzoic acid and its derivative, gallic acid, as the main phenolics. *M. indica* ‘Namdokmai’ also exhibited high inhibitory capacities (>60% inhibition under studied conditions) against lipase, α-amylase and α-glucosidase, key enzymes as drug targets for controlling obesity and type 2 diabetes. Interestingly, all fruit extracts suppressed food mutagen-induced DNA mutations assayed by the Ames test, especially *M. indica* ‘Namdokmai’ and *C. papaya* ‘Khaekdum’ (>50% inhibition at 200 µg/plate). The *M. indica* ‘Namdokmai’ was also the most potent extract for suppression of cancer promotion (>90% inhibition at 200 µg/mL) followed by *P. guajava* ‘Kimju’, *P. guajava* ‘Keenok’ and *C. papaya* ‘Khaekdum’. Results potentially indicated that fruit intake after overcooked meat consumption might supplement nutrients and fiber and also reduce DNA mutation sources.

## 1. Introduction

Non-communicable diseases (NCDs) including cancer, diabetes, hypertension and cardiovascular diseases are leading global causes of public healthcare. Worldwide, new cancer cases in 2020 numbered 19.3 million [1], which is increasing annually, suggesting a rise in cancer prevalence as an important issue facing healthcare systems. However, many studies have supported the beneficial effects of fruit and vegetable intake. The previous cohort studies had suggested that consumption of fruits and vegetables at approximately 5 serving/day could effectively lower risk of mortality [2]. Besides, a systematic review and dose-response meta-analysis from 95 studies revealed that fruit and vegetable consumption correlated with decreased risk of cardiovascular disease, cancers and mortality. The study also revealed that fruit and vegetable intake should exceed 600 g/person/day to reduce cancer risk [3]. Reduction of cancer risk through consumption of fruits and vegetables may result from their phytochemical contents [4]. Furthermore, meta-analysis also showed that fruit consumption, particularly berries, reduced the risk of type 2 diabetes [5]. For NCD prevention, some phytochemicals exhibited enzyme inhibitory activities involved in disease progression, such as lipase, α-amylase and α-glucosidase that are responsible for obesity and diabetes [6].

Fruits and vegetables are well-known sources of minerals, vitamins, dietary fiber and non-nutrient compounds including phytochemicals that are classified into many groups including flavonoids, phytosterols, alkaloids, tannins and carotenoids [7]. Interestingly, most phytochemicals exhibit a wide range of health benefits [8]. Thailand is located in the tropics, and the climate is suitable for growing various fruits. Tropical fruits are rich in phytonutrients with high health benefits. For examples, *Psidium guajava* or guava rich in ellagic acid and rutin exhibits high antioxidant activity [9,10], while *Durio zibethinus* or durian high in luteolin, apigenin, cinnamic acid, and gallic acid provides anti-proliferative and potent cholesterol-lowering activities [11]. Besides, pineapple (*Ananas comosus*) rich in bromelain possesses many potential health benefits such as antiproliferative properties against colorectal carcinoma cells, anti-inflammatory, reducing risk of diabetes and cerebro-vascular diseases [12], while *Mangifera indica* or mango high in mangiferin and several phenolics exhibits anti-inflammatory, anti-tumor and neuroprotective effects [13]. Interestingly, *Carica papaya* or papaya with various types of phytochemicals, especially carotenoids, possesses gastroprotective, anti-tumor, anti-sickling, anti-thrombocytopenic, and immunomodulatory activities [14]. However, several studies on the health benefits regarding inhibition against the key enzymes relevant to obesity (lipase) and diabetes (α-amylase and α-glucosidase) are limited to citrus, pomegranate, berry and *Prunus* fruits [15,16]. Besides, the roles of fruit extracts have been intensively studied for their cancer-killing effects or prevention of metastasis, while inhibition of cancer initiation driven by DNA mutations that is a critical step in carcinogenesis remains uninvestigated. Different cultivars should be compared to observe their effects on phytochemical profiles and biological activities. Therefore, this study comprehensively investigated the phytochemical contents, antioxidant activities, enzyme inhibitory activities and antimutagenic (by Ames test) and chemopreventive properties (by phenotypic screening or anti-deforming assay) of some tropical fruits popularly grown in Thailand including *P. guajava* ‘Kimju’, *P. guajava* ‘Keenok’, *A. comosus* ‘Pattavia’, *A. comosus* ‘Phulae’, *D. zibethinus* ‘Chanee’, *D. zibethinus* ‘Monthong’, *C. papaya* ‘Khaekdum’ and *M. indica* ‘Namdokmai’. Even though some of these fruits had been previously investigated regarding their phytochemicals and bioactivities, limited information on the above cultivars was available. Knowledge gained from this study will promote consumption of tropical fruits as rich natural resources of phytochemicals with potential health-promoting properties, which, with further investigation, could lead to prevention of obesity, type II diabetes and cancer.

## 2. Materials and Methods

### 2.1. Sample Preparation and Extraction

Eight fruits including *Psidium guajava* ‘Kimju’, *Psidium guajava* ‘Keenok’, *Ananas comosus* ‘Pattavia’, *Ananas comosus* ‘Phulae’, *Durio zibethinus* ‘Chanee’, *Durio zibethinus* ‘Monthong’, *Carica papaya* ‘Khaekdum’ and *Mangifera indica* ‘Namdokmai’ were purchased from Simummuang Market, Lam Luk Ka District, Pathum Thani Province, Thailand. The fruit samples and their ripening stages were identified and authenticated by Assoc. Prof. Dr. Chusri Trisonthi (Taxonomist, Faculty of Science, Chiang Mai University, Chiang Mai, Thailand) according to the reliable fruit references [17,18]. Physical appearances of the fruits are shown in Supplementary Table S1. Fresh samples were peeled and cleaned with deionized water before separating the flesh from the seeds (if any). Clean samples were cut into small pieces (approximately 0.5 cm thick), freeze-dried for 3 days using a Super Modulyo-230 freeze dryer (Thermo Fisher Scientific, Waltham, MA, USA), and ground using a Philips 600W grinder (Philips Electronics Co., Ltd., Jakarta, Indonesia) into fine powder. Moisture contents of the dry samples were analyzed by a Halogen HE53 moisture analyzer (Mettler-Toledo AG, Greifensee, Switzerland) and were below 10%. The powdery samples were kept at −20 °C until required for further analysis.

Extraction of the fruit samples was performed as previously described [19] with some modifications as follows. Powdered samples (100 g) were extracted using a solvent mixture comprising methanol, acetone and water in a ratio of 2:2:1 (400 mL) for 24 h. Filtrates were obtained using a Büchner funnel filtration set equipped with a Whatman no.54 filter paper and an aspirator pump. Solvent removal was conducted by a rotary vacuum evaporator (Büchi Corporation, New Castle, DE, USA) with water bath temperature set at 40 °C. Removal of water-soluble components from crude extracts was conducted using a solid phase extraction (SPE) Sep-Pak C18 cartridge (Waters Corporation, Milford, MA, USA). The cartridge (5 g) was rinsed with methanol followed by distilled water twice (50 mL each). Dried crude extracts were dissolved in distilled water (500 mL), assisted with ultrasonic agitation. Then the extract was pumped into a pre-rinsed cartridge and eluted with distilled water (100 mL) with a flow rate of 5 mL/min. The filtrate was then re-extracted by liquid-liquid extraction using ethyl acetate (25 mL) to collect the remaining water-insoluble components (fraction 1) and discard the remaining water-soluble compounds, which cannot be moved to ethyl acetate. Finally, the solid phase attached within the cartridge was eluted using a mixture of methanol and acetone (1:1) (100 mL). The obtained eluate (fraction 2) was combined with the ethyl acetate fraction (fraction 1), and all solvents were removed using the rotary vacuum evaporator. The dried extract was stored at −20 °C until required for use.

### 2.2. Determination of Phytochemicals

To determine phenolic profiles, the acidic methanol extraction and high-performance liquid chromatography (HPLC) were employed and validated as previously described [20,21]. The phenolics were identified and quantitated utilizing an Agilent 1100 HPLC equipped with a photodiode array detector and a Zorbax Eclipse XDB-C_18_ column (5 µm, 150 × 4.6 mm) from Agilent Technologies (Santa Clara, CA, USA). A gradient mobile phase consisted of solvent A: Milli-Q water (18.2 MΩ.cm resistivity at 25 °C) containing 0.05% (*v*/*v*) trifluoroacetic acid (TFA), solvent B: methanol containing 0.05% (*v*/*v*) TFA, and solvent C: acetonitrile containing 0.05% (*v*/*v*) TFA), and a constant flow rate of 0.6 mL/min. The phenolic standards were apigenin (>98.0% HPLC), caffeic acid (>98.0% HPLC, T), chlorogenic acid (>98.0% HPLC, T), *p*–coumaric acid (>98.0% GC, T), 3,4–dihydroxybenzoic acid (≥97% T), ferulic acid (>98.0% GC, T), genistein (>98.0% HPLC), hesperidin (>90.0% HPLC, T), 4–hydroxybenzoic acid (>99.0% GC, T), kaempferol (>97.0% HPLC), luteolin (>98.0% HPLC), myricetin (>97.0% HPLC), naringenin (>93.0% HPLC, T), quercetin (>98.0% HPLC, E), syringic acid (>97.0% T), and sinapic acid (>99.0% GC, T), which were obtained from Tokyo Chemical Industry (Tokyo, Japan). Gallic acid (97.5–102.5% T), vanillic acid (≥97% HPLC), and rosmarinic acid (≥98% HPLC) were received from Sigma–Aldrich (St. Louis, MO, USA), while isorhamnetin (≥99.0% HPLC) was obtained from Extrasynthese (Genay, France). The phenolics were visualized at 280, 325, 338, and 368 nm. The HPLC chromatograms were shown in Supplementary Figures S1–S4.

Total phenolic contents (TPCs) were determined as previously described [22,23] with some modifications as follows. The fruit extracts (25 µL) in 10% (*v*/*v*) dimethyl sulfoxide (DMSO) were mixed with 10% (*v*/*v*) Folin-Ciocalteu reagent (50 μL) and incubated for 5 min. To the mixture, 7.5% (*w*/*v*) saturated sodium bicarbonate (200 μL) was added and mixed well. The mixture was then incubated in dark at room temperature (25 °C) for 2 h. The TPCs were measured at 765 nm using a Synergy^TM^ HT 96–well UV–visible microplate reader (BioTek Instruments, Inc., Winooski, VT, USA) with a Gen 5 data analysis software. Gallic acid in a range of 0–200 µg/mL was used as a standard, and the TPCs were expressed as mg gallic acid equivalents (GAE)/g extract.

Total flavonoid contents (TFCs) were analyzed according to a well-established protocol [22] with some modifications as follows. The fruit extracts in 10% (*v*/*v*) DMSO (165 µL) were mixed with 5% (*w*/*v*) sodium nitrite (9 μL). After 6 min of incubation, 10% (*w*/*v*) aluminum chloride hexahydrate (18 μL) was added, and incubated for another 5 min. To the mixture, 1 M sodium hydroxide (60 µL) was added, and the TFCs were measured at 510 nm using the 96–well UV–visible microplate reader. Quercetin at the concentration ranging 0–100 µg/mL was used as a standard, and the TFCs were expressed as mg quercetin equivalent (QE)/g extract.

### 2.3. Determination of Antioxidant Activities

The antioxidant activities were performed using 2,2-diphenyl-1-picrylhydrazyl (DPPH) radical scavenging, ferric ion reducing antioxidant power (FRAP), and oxygen radical absorbance capacity (ORAC) assays as previously described [23]. The dried extracts were dissolved in 10% (*v*/*v*) DMSO before analyzing antioxidant activities. The DPPH radical scavenging activity was evaluated using the DPPH free radical solution, a Trolox standard in the concentrations of 0.01–0.64 mM, and a detection at 520 nm. The FRAP activity was determined using FRAP reagent, the Trolox standard of 7.81–250.00 µM, and a detection at 595 nm. Lastly, the ORAC activity was determined using a fluorescein reagent, the Trolox standard of 3.12–100.00 µM, and the detection at an excitation wavelength of 485 nm and emission wavelength of 528 nm. All assays were performed on the 96–well UV–visible microplate reader and expressed as µmol Trolox equivalent (TE)/g extract.

### 2.4. Determination of Enzyme Inhibitory Activities

The inhibitory activities were determined using the key enzymes relevant to obesity (lipase) and diabetes (α-amylase and α-glucosidase). The enzyme assays were performed using the well-established protocols as previously described [6,24] on the 96–well UV–visible microplate reader. The dried extracts were dissolved in 10% (*v*/*v*) DMSO before performing enzyme inhibitory assays. All chemicals and reagents were received from Sigma-Aldrich (St. Louis, MO, USA).

The lipase inhibitory assay was performed using 100 µL of 5 µg/mL *Candida rugosa* lipase (type VII, ≥700 unit/mg), 10 µL of 16 mM 5,5′-dithiobis(2-nitrobenzoic acid), 50 µL of 0.2 mM 5-5′-dithiobis(2-nitrobenzoic-*N*-phenacyl-4,5-dimethyyhiazolium bromide) and 40 µL of the extract. The inhibitory activities were detected at 412 nm.

The α-amylase inhibitory assay was performed using 100 µL of 20 mg/mL porcine pancreatic α-amylase (type VII, ≥10 unit/mg), 50 µL of 30 mM *p*-nitrophenyl-α-D-maltopentaoside and 50 µL of the extract. The α-glucosidase inhibitory assay was performed using 100 µL of 1 U/mL *Saccharomyces cerevisiae* α-glucosidase (type I, ≥10 unit/mg), 50 µL of 2 mM *p*-nitrophenyl-α-D-glucopyranoside and 50 µL of the extract. The inhibitory activities were detected at 405 nm.

The inhibitory activities were expressed as percentage of inhibition using the following equation:

(1) $$\mathrm{Percentage}\;(\%)\;\mathrm{of}\;\mathrm{inhibition}=100\;\times \;\left(1-\frac{B-b}{A-a}\right)$$

where *A* is the initial velocity of the control reaction with enzyme (control), *a* is the initial velocity of the control reaction without enzyme (control blank), *B* is the initial velocity of the enzyme reaction with extract (sample), and *b* is the initial velocity of the reaction with extract but without enzyme (sample blank).

### 2.5. Determination of Mutagenicity and Anti-Mutagenicity Using Ames Test

Mutagenicity testing was performed using *Salmonella typhimurium* tester strain, TA98, which is sensitive to frameshift mutations, as previously described [25]. In brief, the fruit extracts (100–300 mg/plate) were mixed with TA98 (provided by Dr. W. Kusamran from the National Cancer Institute, Ministry of Public Health, Bangkok, Thailand) at 2 × 10^9^ CFU/mL, phosphate buffer and S9 mix (mouse liver homogenate, Sigma-Aldrich, St. Louis, MO, USA). The contents were then mixed with top agar and poured on minimal agar plates followed by incubation at 37 °C. After 48 h, the number of histidine revertant colonies of each plate was counted, and 2-aminofluorene (2-AF, 1 µg/plate) was used as a positive control. All experiments were performed at least in triplicate.

Anti-mutagenicity testing was performed using the active growth of TA98 at 2 × 10^9^ CFU/mL. This was mixed with each indirect standard mutagen, including tryptophan pyrolysis products (Trp-P1 at 50 ng/plate or Trp-P2 at 20 ng/plate) and 2-amino-3,8-dimethylimidazo [4,5-f]quinoxaline (MeIQx) at 25 ng/plate, phosphate buffer and S9 mix. The contents were then mixed with top agar and poured on minimal agar plates followed by incubation at 37 °C for 48 h. Then, the number of histidine revertant colonies on each plate was determined. The percentage of inhibition was calculated using the following equation:

(2) $$\mathrm{Percentage}\;(\%)\;\mathrm{of}\;\mathrm{inhibition}=100\;\times \;\left(\frac{C0\;-S}{C0\;-\;C100}\right)$$

where *C*_0_ is the number of revertant colonies from the positive control per plate, *C*_100_ is the number of spontaneous revertant colonies from the negative control per plate, and *S* is the number of revertant colonies per plate induced by mutagen in the presence of the fruit extract. Percentage of inhibition was strong when it was higher than 60%, 60–41% was moderate, 40–21% was weak, and <20% had a negligible effect [26].

### 2.6. Determination of Chemopreventive Properties Using Phenotypic Screening Assay or Anti-Deforming Assay

Human Burkitt lymphoma cells (Raji, ATCC no.CCL-86) were cultured in Roswell Park Memorial Institute 1640 (RPMI 1640) (Gibco^®^, Thermo Fisher Scientific, Waltham, MA, USA), containing 10% (*v*/*v*) fetal calf serum (Hyclone, Marlborough, MA, USA), pyruvate (1 mM), penicillin (100 U/mL) and streptomycin (100 µg/mL) at 37 °C in a 5% CO_2_ incubator (Galaxy^®^ 48 R, Eppendorf, Hamburg, Germany).

Cytotoxicity was analyzed using a water-soluble tetrazolium salt (WST-1) assay. Concentrations of 1 × 10^4^ exponentially growing Raji cells were seeded in RPMI 1640 and cultured per well of a 96-well plate for 24 h. Before testing, cells were washed with phosphate buffer saline (PBS) and treated with the fruit extract up to 200 µg/mL. After 48 h, tetrazolium salt from the Cell Proliferation Reagent WST-1 kit (Merck, Darmstadt, Germany) was added and incubated at 37 °C in the dark for an hour. Then, the amount of cell viability, which was represented by the amount of formazan formation, was determined using a microplate reader (Infinite^®^ 200 PRO, Tecan, Männedorf, Switzerland) at 440 nm.

Screening of chemopreventive properties was determined using a phenotypic screening assay. Concentrations of 1 × 10^5^ log phase cells were cultured per well of a 6-well plate in RMPI 1640 containing 10% (*v*/*v*) fetal calf serum, pyruvate (1 mM), penicillin (100 U/mL), streptomycin (100 µg/mL), 10 mM sodium butyrate and 65 nM 12-*O*-tetradecanoyl-phorbol-13-acetate (TPA or PMA, Merck, Darmstadt, Germany) with or without the fruit extract as described [27]. Cells were then incubated for the next 48 h at 37 °C. Deformed Raji cells (tree branch-like, dilation and flatness) were scored under a phase contrast microscope with a DP74 camera (IX83, Olympus, Tokyo, Japan). Counting was conducted randomly from ten areas covering at least 500 cells.

### 2.7. Statistical Analysis

All experiments were performed in triplicate (*n* = 3) or as indicated otherwise, and the results were expressed as mean ± standard deviation (SD). The significant differences between values were determined at *p* < 0.05 using one–way analysis of variance (ANOVA), followed by Duncan’s multiple comparison test (more than two data) or Student’s unpaired *t*-test (two data) from the statistical package for the social sciences (version 18 for Windows, SPSS Inc., Chicago, IL, USA).

Principal component analysis (PCA) and hierarchical cluster analysis (HCA) of TPCs, antioxidant activities, and enzyme inhibitory activities were determined using XLSTAT^®^ Trial (Addinsoft Inc., New York, NY, USA).

## 3. Results

### 3.1. Phytochemical Analysis

Phenolic profiles including phenolic acids and flavonoids of eight fruit extracts including *Psidium guajava* ‘Kimju’, *Psidium guajava* ‘Keenok’, *Ananas comosus* ‘Pattavia’, *Ananas comosus* ‘Phulae’, *Durio zibethinus* ‘Chanee’, *Durio zibethinus* ‘Monthong’, *Carica papaya* ‘Khaekdum’ and *Mangifera indica* ‘Namdokmai’ were analyzed using high-performance liquid chromatography (HPLC) (Table 1). Results indicated that among phenolic acids, the highest amounts of gallic acid and 4-hydroxybenzoic acid were detected in *M. indica* ‘Namdokmai’, while the highest ferulic acid content was detected in *A. comosus* ‘Pattavia’. The highest content of caffeic acid was detected in *A. comosus* ‘Phulae’, and *p*-coumaric acid in *D. zibethinus* ‘Chanee’. The fruit extract of *P. guajava* ‘Kimju’ possessed the highest contents of syringic acid and chlorogenic acid, while the highest vanillic acid content was found in *D. zibethinus* ‘Monthong’. Sinapic acid was only detected in *C. papaya* ‘Khaekdum’. Among the fruit extracts, *C. papaya* ‘Khaekdum’ exhibited the greatest variety of phenolic acids, while only one phenolic acid was detected in *P. guajava* ‘Keenok’. *A. comosus* ‘Phulae’ contained the greatest variety of flavonoids with hesperidin the highest. *A. comosus* ‘Phulae’ contained the highest contents of naringenin and apigenin, while kaempferol was only detected in this extract. Luteolin was mostly found in *A. comosus* ‘Pattavia’, with quercetin the highest in *P. guajava* ‘Kimju’. Trace amounts of isorhamnetin were only found in both cultivars of *P. guajava*, while myricetin was only detected in both cultivars of *A. comosus*. Interestingly, no flavonoid was detected in both cultivars of *D. zibethinus*.

Total phenolic contents (TPCs) of all fruit extracts ranged 2.67–94.75 mg gallic acid equivalent (GAE)/g extract with the highest detected in *M. indica* ‘Namdokmai’ and the lowest in *C. papaya* ‘Khaekdum’ (Table 1). Interestingly, total flavonoid contents (TFCs) ranging 8.43–286.82 mg quercetin equivalent (QE)/g extract were the highest in *D. zibethinus* ‘Chanee’ and the lowest in *A. comosus* ‘Phulae’ (Table 1).

Comparing between cultivars, *P. guajava* ‘Kimju’ exhibited greater varieties and quantities of phenolics than ‘Keenok’ cultivar, leading to greater TPCs and TFCs (1.2 and 2.5 times greater, respectively). Even though *A. comosus* ‘Phulae’ exhibited greater varieties of phenolics than ‘Pattavia’ cultivar, the later exhibited 3.2 and 5.1 times higher TPCs and TFCs, respectively than the former. Similar results were observed in *D. zibethinus*, whereby ‘Monthong’ cultivar exhibited a greater variety of phenolics but lower contents than ‘Chanee’, which exhibited 1.3 and 28.7 times higher TPCs and TFCs, respectively than ‘Monthong’ cultivar.

### 3.2. Antioxidant Activities

Antioxidant activities were performed using 2,2-diphenyl-1-picrylhydrazyl (DPPH) radical scavenging, ferric reducing antioxidant power (FRAP) and oxygen radical antioxidant capacity (ORAC) assays to investigate different antioxidant mechanisms of the fruit extracts. The DPPH radical scavenging and FRAP assays follow single electron transfer (SET) mechanism, while the ORAC assay follows the hydrogen atom transfer (HAT) mechanism.

Results indicated that all fruit extracts possessed DPPH radical scavenging activities in the range 0.04–0.95 μmol Trolox equivalent (TE)/g extract, FRAP activities in the range 76.24–691.36 μmol TE/g extract, and ORAC activities in the range 248.37–1266.82 μmol TE/g extract (Table 2). Among the fruit extracts, *M. indica* ‘Namdokmai’ exhibited the highest antioxidant activities measured by all three methods followed by *P. guajava* ‘Kimju’, *P. guajava* ‘Keenok’, *A. comosus* ‘Pattavia’, *A. comosus* ‘Phulae’, *D. zibethinus* ‘Chanee’, *D. zibethinus* ‘Monthong’ and *C. papaya* ‘Khaekdum’.

Comparing between cultivars, *P. guajava* ‘Kimju’ exhibited 1.0 to 1.1 times higher antioxidant capacities than ‘Keenok’ cultivar, while *A. comosus* ‘Pattavia’ exhibited 2.0 to 3.4 times greater antioxidant capacities than ‘Phulae’ cultivar. Antioxidant capacity in *D. zibethinus* ‘Chanee’ was 1.1 to 2.1 times greater than ‘Monthong’ cultivar. These results suggested that fruit extracts with high TPCs exhibited high antioxidant capacities.

### 3.3. Enzyme Inhibitory Activities

Inhibitory activities of the key enzymes relevant to obesity (lipase) and diabetes (α-amylase and α-glucosidase) were tested to investigate the potential biological functions of the fruit extracts. Lipase, a lipid degrading enzyme, is one of the anti-obesity drug targets to control the availability of excessive fat before absorption into the body. Likewise, α-amylase and α-glucosidase are also targeted to control diabetes since they are carbohydrate degrading enzymes.

Results indicated that lipase inhibitory activities ranged 25.09–77.12% using extract concentration of 0.05 mg/mL (Table 3). The fruit extract of *A. comosus* ‘Pattavia’ exhibited the highest lipase inhibitory activity, while no activity was observed in *P. guajava* ‘Kimju’, *P. guajava* ‘Keenok’ and *C. papaya* ‘Khaekdum’. Comparing between cultivars, *A. comosus* ‘Pattavia’ exhibited 2.4 times higher lipase inhibitory activity than ‘Phulae’ cultivar, while inhibition in *D. zibethinus* ‘Chanee’ was 2.9 times higher than ‘Monthong’ cultivar. These results suggested that the fruit extracts with high TPCs also exhibited high lipase inhibitory activities.

Interestingly, all fruit extracts exhibited α-amylase inhibitory activities ranging 5.36–68.00% using extract concentration of 0.1 mg/mL (Table 3). Among these, *M. indica* ‘Namdokmai’ exhibited the highest inhibitory activities, while *C. papaya* ‘Khaekdum’ and *D. zibethinus* ‘Monthong’ gave the lowest. The α-glucosidase inhibitory activities ranged 17.34–93.95% using extract concentration of 0.1 mg/mL. Again, *M. indica* ‘Namdokmai’ exhibited the highest α-glucosidase inhibitory activity, while no inhibition was observed in the fruit extract of *A. comosus.* Comparing between cultivars, *P. guajava* ‘Keenok’ exhibited 1.4 times higher α-amylase inhibitory activity than ‘Kimju’ cultivar but their α-glucosidase inhibitions were insignificantly different. The α-amylase inhibitory activity in *A. comosus* ‘Pattavia’ was 1.1 times higher than ‘Phulae’ cultivar, while inhibition in *D. zibethinus* ‘Chanee’ was 2.4 times higher than ‘Monthong’ cultivar. However, *D. zibethinus* ‘Monthong’ exhibited 1.2 times higher α-glucosidase inhibitory activity than ‘Chanee’ cultivar. These results suggested that rather than TPCs, types of phenolics might play a significant part in α-amylase and α-glucosidase inhibitory activities.

### 3.4. Mutagenicity and Anti-Mutagenicity Analysis of Fruit Extracts

One hallmark of cancer is genomic instability driven by DNA mutations [28]. Several studies have shown that consumption of vegetables and fruits might be associated with a reduction in cancer risk [29,30], due to their rich nutritive values and phytochemicals that play a role as antimutagenic agents [31]. As shown in Table 1, all eight fruit extracts contained various phytochemicals; thus, both the mutagenic and antimutagenic properties of the extracts were tested using the *Salmonella typhimurium*/microsome assay (Ames test) to determine their mutagenic and antimutagenic potentials. Advantages of this assay include rapid and representative metabolic activation with the addition of liver homogenate (S9 mix). *S. typhimurium* strain TA98 was used as a bacterial model. Results indicated that TA98 displayed a spontaneous background of revertant colonies (negative control) in the presence of the S9 mix ranging from 25–37 colonies (Table 4), and in the normal range of this strain [32]. The revertant colonies numbered 1252 after exposure to 2-aminofluorene (2-AF, a potent mutagen), implying that the tested strain was sensitive to detect mutagens with metabolic activation. Three extract concentrations (100, 200 and 300 mg/plate) that did not alter bacterial growth (data not shown) were used in the assay. Data showed no induction of revertant colonies when TA98 was exposed to all ranges of fruit extracts in the presence of metabolic activation, while dose-dependent was absent (Table 4), indicating that the fruit extracts were not mutagenic in this condition.

**Figure 1:**
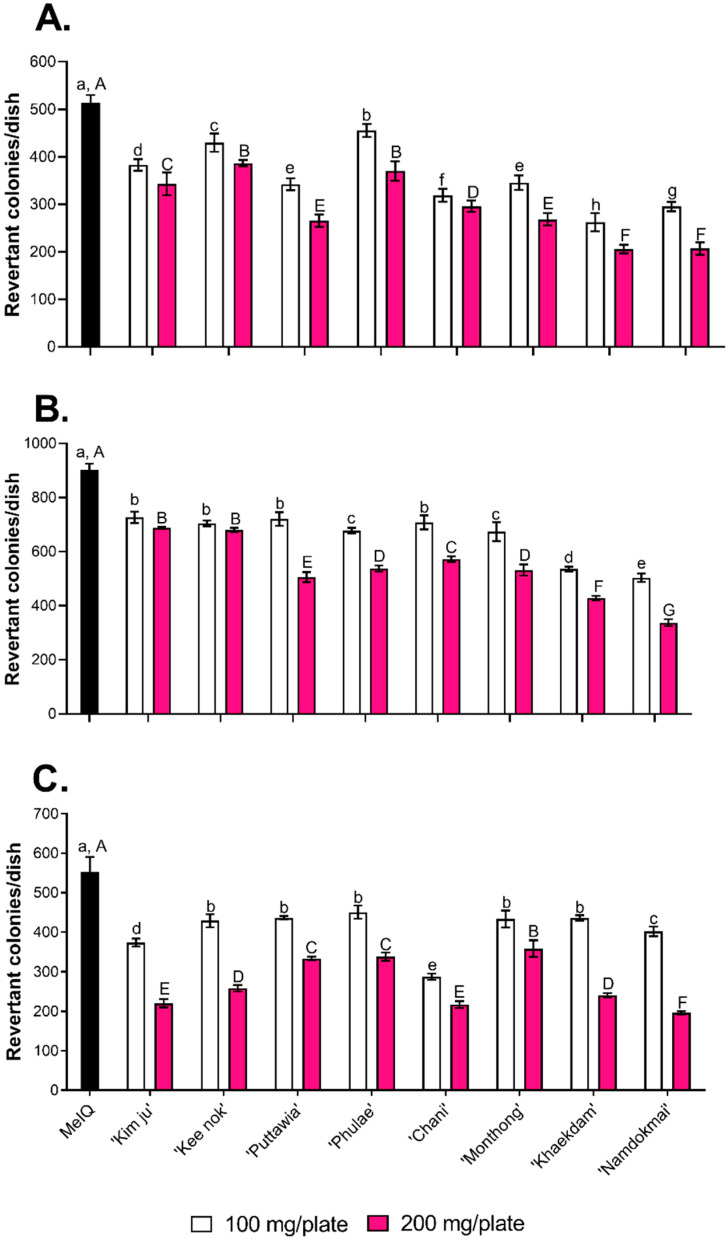
Effect of the eight fruit extracts on inhibition of chemical-mediated DNA mutation determined by TA98 in the presence of S9 mix. (**A**) Inhibition against 3-amino-1,4 dimethyl-5H-pyrido[4,3 b]indole (Trp-P1), (**B**) Inhibition against 3-amino-1-methyl-5H-pyrido[4.3-b]indole (Trp-P2), and (**C**) Inhibition against 2-amino-3,4-dimethylimidazo[4,5-ƒ]quinoline (MeIQ). Different small letters indicate significant differences (*p* < 0.05) among fruit extracts at 100 mg/plate compared to each positive control, while different capital letters indicate significant differences (*p* < 0.05) among fruit extracts at 200 mg/plate compared to each positive control. Error bars represent standard deviations (SD) from the four experiments.

Mutagenicity testing indicated the preliminary safety of the fruit extracts and their anti-mutagenicity against three mutagens including (i) 3-amino-1,4-dimethyl-5H-pyrido [4,3 b]indole (Trp-P1), (ii) 3-amino-1-methyl-5H-pyrido[4.3-b]indole (Trp-P2), and (iii) 2-amino-3,4-dimethylimidazo[4,5-ƒ]quinoline (MeIQ). All mutagens were potent food-borne heterocyclic amines found in cooked foods [33]. The Ames test with TA98 was again used as a model. Figure 1A–C shows the number of revertant colonies as 513, 902 and 553 after exposure to Trp-P1, Trp-P2 and MeIQ, respectively, confirming their potent mutagenicity. Figure 1A–C also shows that all eight fruit extracts significantly reduced revertant colonies compared to each positive control in the presence of mutagen, extract and S9 mix, albeit with different abilities. The extracts of *C. papaya* ‘Khaekdum’ and *M. indica* ‘Namdokmai’ effectively inhibited DNA mutation induced by both Trp-P1 and Trp-P2 (Figure 1A,B), while high doses of *P. guajava* ‘Kimju’, *P. guajava* ‘Keenok’, *D. zibethinus* ‘Chanee’, *C. papaya* ‘Khaekdum’ and *M. indica* ‘Namdokmai’ effectively suppressed MeIQ-mediated DNA mutations (Figure 1C).

### 3.5. Chemopreventive Properties of Fruit Extracts

DNA mutation is an initiation step in carcinogenesis. As shown in Figure 1, all fruit extracts reduced chemical-mediated DNA mutations. Hence, it was interesting to examine whether the extracts exhibited chemopreventive properties. Here, the phenotypic screening assay or anti-deforming assay in Raji cells was utilized. Raji cells are human lymphoblastoid cells with Epstein Barr Virus (EBV) early antigen (EA) integrated into the genome [34]. Under normal conditions, the EA is not expressed, leading to unchanged cell characteristics. Tumor-promoting agents like 12-*O*-tetradecanoylphorbol13-acetate (TPA) and sodium butyrate induce EA-mediated morphological changes or deformed cells. These morphological changes (tree branch-like, dilation and flatness) render cancer-like Raji cells. Supplementary Figure S5 shows the cell morphology of Raji cells under both positive and negative conditions. Hence, inhibition of Raji morphological changes was used as a readout for inhibitory properties of cancer promotion of the extracts [34].

**Figure 2:**
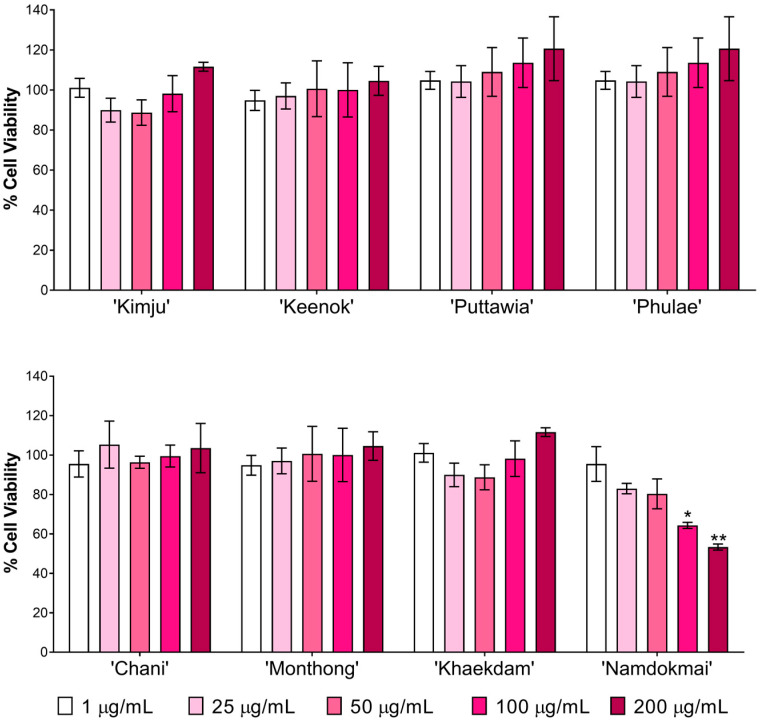
Effect of the eight fruit extracts on cell viability of human Burkitts lymphoma cells (Raji) determined by the water-soluble tetrazolium salt (WST-1) assay. Data are expressed as mean ± standard deviation (SD) of three experiments. The percentage of cell viability (% cell viability) was evaluated, and statistical significance was analyzed by Student’s unpaired *t*-test against its control group. *, *p* < 0.05 and **, *p* < 0.01.

**Figure 3:**
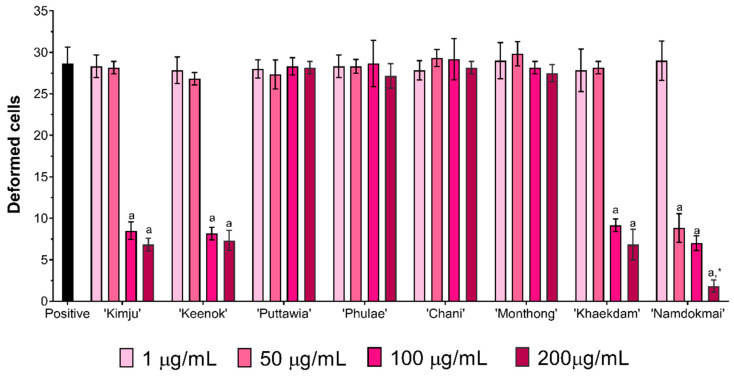
Effect of the eight fruit extracts on deformation of Raji cells after exposure to TPA and sodium butyrate. Numbers of deformed cells were counted from a total of 500 cells. Different lower case letters indicate significant differences (*p* < 0.05) between each concentration of the extract compared to the positive control. The asterisk (*) indicates the significance of this dose within *Mangifera indica* ‘Namdokmai’. Error bars represent standard deviations (SD) from four experiments.

**Figure 4:**
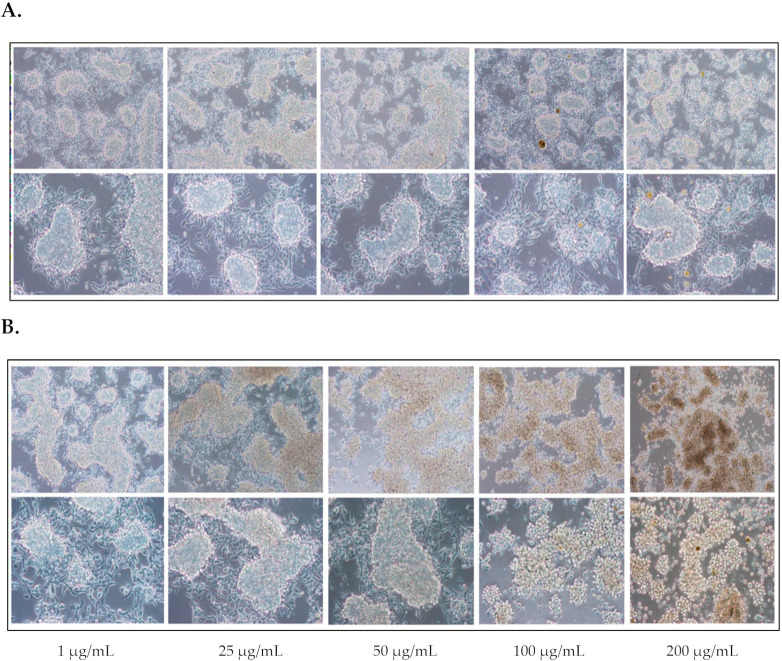
Cell morphology of Raji cells after treatment with 0.1% (*v*/*v*) dimethyl sulfoxide (DMSO), 1 mM sodium butyrate, 1 mM 12-*O*-tetradecanoylphorbol13-acetate (TPA) and extracts of (**A**) *Ananas comosus* ‘Phulae’ or (**B**) *Mangifera indica* ‘Namdokmai’ from 1–200 µg/mL. Upper picture magnification: 20× and lower picture magnification: 40×.

First, the cytotoxicity of the fruit extracts toward Raji cells was studied using a water-soluble tetrazolium salt (WST-1) assay. The cells were treated with various concentrations of fruit extracts (0–200 µg/mL) for 48 h. Results indicated that, except for *M. indica* ‘Namdokmai’ at 100 and 200 µg/mL, fruit extracts were not cytotoxic to Raji cells (Figure 2). Chemopreventive properties of the fruit extracts were further investigated via phenotypic screening assay. In the positive control, Raji cells were deformed at 28 cells from 500 cells after treatment with cancer-promoting compounds, TPA and sodium butyrate (Figure 3). These results concurred with a previous report [27]. Compared to the positive control, *A. comosus* ‘Pattavia’, *A. comosus* ‘Phulae’, *D. zibethinus* ‘Chanee’ and *D. zibethinus* ‘Monthong’ exhibited the same quantity of deformed cells as a positive control, suggesting that these four extracts lacked chemopreventive properties determined by this assay. Interestingly, *P. guajava* ‘Kimju’, *P. guajava* ‘Keenok’, *C. papaya* ‘Khaekdum’ and *M. indica* ‘Namdokmai’ significantly inhibited deformation of Raji cells. Although we did not obtain a dose-dependent effect, the data implied the chemopreventive effect of the tested fruit extracts. *M. indica* ‘Namdokmai’ exhibited cytotoxicity at 100 and 200 µg/mL; therefore, at these doses we were unable to exclude the possibility of cell death effect. However, a safe dose of *M. indica* ‘Namdokmai’ at 50 µg/mL (Figure 3 and Figure 4) strongly inhibited the formation of deformed cells, indicating that among the eight extracts, *M. indica* ‘Namdokmai’ was the most potent inhibitor for cancer promotion. Figure 4 shows an example of Raji cells treated with TPA and *A. comosus* ‘Phulae’ or *M. indica* ‘Namdokmai’, while Supplementary Figure S6 shows representative images of Raji cells treated with other fruit extracts.

### 3.6. Correlation by Principal Component Analysis (PCA) and Hierarchical Cluster Analysis (HCA)

To determine the relationship between fruit extracts and TPCs, antioxidant properties (DPPH radical scavenging, FRAP and ORAC activities) and enzyme inhibitory activities (lipase, α-amylase and α-glucosidase), principal component analysis (PCA) and hierarchical cluster analysis (HCA) were employed. These techniques are suitable for the evaluation of various factors within one step compared to the Pearson correlation.

**Figure 5:**
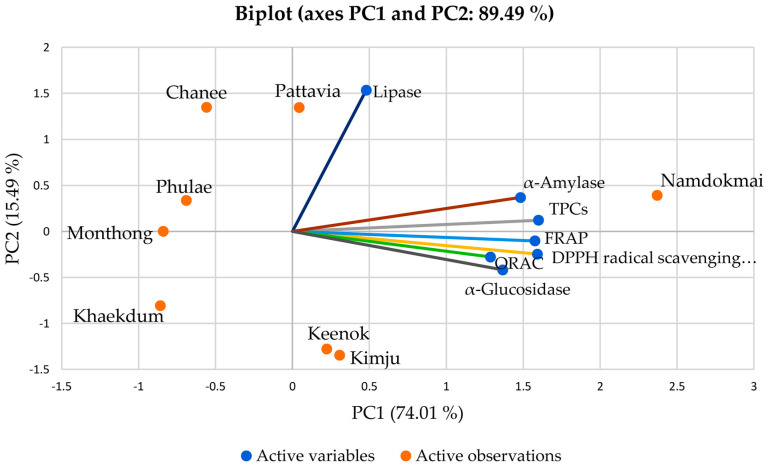
The biplot from principal component analysis (PCA) from mean values of all variables of the eight fruit extracts.

After the first PCA analysis, TFCs were separated from the total variables determined by Bartlett’s sphericity test (data not shown), indicating that TFCs did not correlate with all variables. Thus, the second analysis excluded TFCs from the PCA analysis. Figure 5 illustrates the relationship among observations (taxa of fruit extracts), variables (TPCs, DPPH radical scavenging activities, FRAP activities, ORAC activities, lipase inhibitory activities, α-amylase inhibitory activities and α-glucosidase inhibitory activities) as a biplot (combined between observations and variables). The first two axes (PC1 and PC2) covered 89.49% of the total variables. PC1 (74.04%) contained TPCs, DPPH radical scavenging activities, FRAP activities, ORAC activities, α-amylase inhibitory activities and α-glucosidase inhibitory activities, while PC2 (15.49%) contained only lipase inhibitory activities. Interestingly, *M. indica ‘Namdokmai’*, *P. guajava* ‘Kimju’ and *P. guajava* ‘Keenok’ were located in PC1, indicating that these three extracts exhibited high TPCs, DPPH radical scavenging activities, FRAP activities, ORAC activities, α-amylase inhibitory activities and α-glucosidase inhibitory activities. On the other hand, *A. comosus* ‘Pattavia’, *A. comosus* ‘Phulae’, *D. zibethinus* ‘Chanee’ and *M. indica* ‘Namdokmai’ were projected in PC2, indicating that they were potent anti-lipase inhibitors. *M. indica* ‘Namdokmai’ was the only extract projected in both PCs, and it was the most potent fruit extract against antioxidant, obesity and diabetes, possibly due to its high TPCs. Interestingly, *D. zibethinus* ‘Chanee’ and *D. zibethinus* ‘Monthong’ were located in PC2 but in the opposite direction, indicating that different cultivars could affected biological activities, particular lipase inhibition.

**Figure 6:**
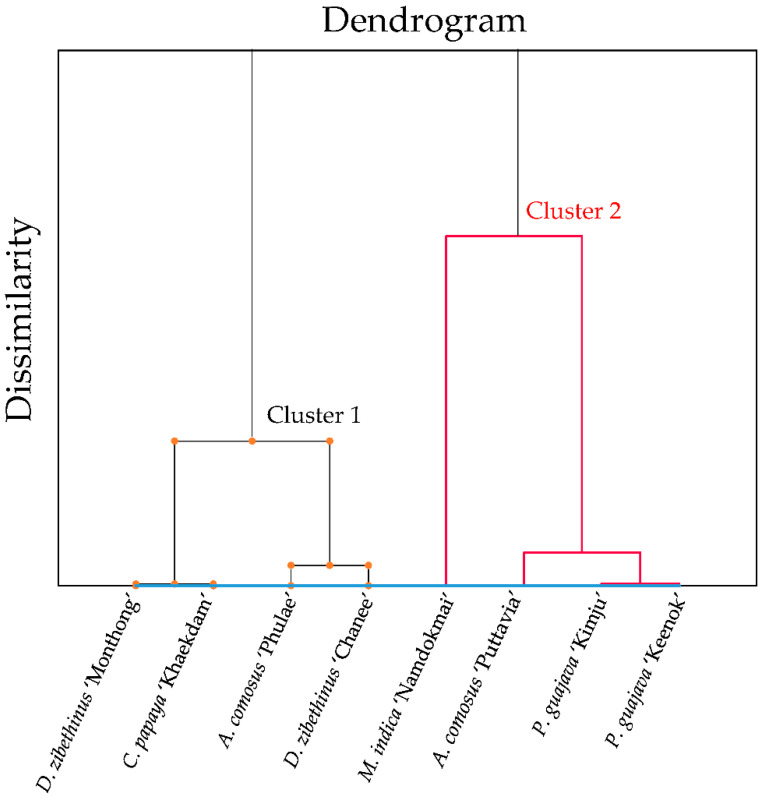
The dendrogram of hierarchical cluster analysis (HCA) of eight tropical fruit extracts.

To further evaluate the extracts, the HCA was employed as shown in Figure 6. The vertical axis represents dissimilarity between the groups. If the values are large, they are considered to be in different groups. The HCA was divided into two groups. Cluster 1 consisted of *A. comosus* ‘Phulae’, *D. zibethinus* ‘Chanee’, *D. zibethinus* ‘Monthong’ and *C. papaya* ‘Khaekdum’, while cluster 2 contained *P. guajava* ‘Kimju’, *P. guajava* ‘Keenok’, *A. comosus* ‘Pattavia’, *D. zibethinus* ‘Chanee’ and *M. indica* ‘Namdokmai’. Both HCA and PCA were well-correlated. Cluster 1 represented extracts harboring moderate to poor activities, while cluster 2 contained extracts with moderate to high activities.

## 4. Discussion

Non-communicable diseases (NCDs) such as cancer, diabetes, hypertension and cardiovascular diseases are critical health problems with long-term risk effects including smoking, alcohol consumption, physical inactivity and especially, unhealthy diets [35]. These factors generate oxidative stress, which plays a significant role in NCD progression [36]. Fruit and vegetable consumption has been proven to ameliorate NCD morbidity and mortality, with reduced side effects compared to synthetic drugs [37]. These health properties are generated by phytochemicals such as polyphenolic compounds, tocopherols and carotenoids [38]. Investigation of the phytochemicals showing antioxidant activities, enzyme inhibitory activities, antimutagenic and chemopreventive properties of eight tropical fruit extracts including *Psidium guajava* ‘Kimju’, *Psidium guajava* ‘Keenok’, *Ananas comosus* ‘Pattavia’, *Ananas comosus* ‘Phulae’, *Durio zibethinus* ‘Chanee’, *Durio zibethinus* ‘Monthong’, *Carica papaya* ‘Khaekdum’ and *Mangifera indica* ‘Namdokmai’ indicated that (i) *M. indica* ‘Namdokmai’ exhibited the highest total phenolic contents (TPCs) with 4-hydroxybenzoic acid and gallic acid the most abundant phenolics, (ii) high TPCs in *M. indica* ‘Namdokmai’ led to high antioxidant activities and enzyme inhibitory potentials, (iii) all tested tropical fruit extracts were not genotoxic and acted as anti-mutagenic compounds against known mutagens, and (iv) *M. indica* ‘Namdokmai’ was the most potent extract in suppression of cancer promotion.

Phenolics found in *M. indica* ‘Namdokmai’ were mainly 4-hydroxybenzoic acid and gallic acid. Results concurred with a previous study on *M. indica* L. cv. Ataulfo, suggesting that gallic acid was also a predominant phenolic (39%), while smaller amounts of 4-hydroxybenzoic acid were detected [39]. Five commercially available mango cultivars including Kent, Tommy Atkins, Keitt, Haden and Ataulfo from Mexico, Peru, Brazil and Ecuador exhibited TPCs ranging from 19.5 to 130.8 mg gallic acid equivalent (GAE)/100 g fresh weight (FW) [40,41]. Interestingly, TPCs tended to increase with longer harvesting time or when the mango ripened [39,40]. Thus, high TPCs detected in *M. indica* ‘Namdokmai’ might be due to its ripening stage.

High content of phenolics, especially gallic acid, led to high antioxidant activities detected in *M. indica* ‘Namdokmai’. Gallic acid is a strong antioxidant, and possesses 2,2–diphenyl–1–picrylhydrazyl (DPPH) radical scavenging activity with half-maximal scavenging concentration (EC_50_) of 140 µmol/mL, ferric ion reducing antioxidant power (FRAP) activity of 320 µmol Trolox equivalent (TE)/moles antioxidant, and oxygen radical absorbance capacity (ORAC) activity of 1.15 mol TE/moles antioxidant [42]. A computational approach suggested that the antioxidant function of gallic acid could be explained using hydrogen atom transfer (HAT) and transition metal chelation (TMC) mechanisms [43].

High phenolics in *M. indica* ‘Namdokmai’ also led to high lipase inhibitory activities. A previous study on seven *M. indica* varieties including Amrapali, Fazli, Golapkhas, Gopalbhog, Himsagar, Langra and Mohanbhog from India suggested that their methanolic extracts exhibited lipase inhibition, with half-maximal inhibitory concentration (IC_50_) ranging 1.5–5.2 mg/mL [44]. The phenolics detected in these mangoes as gallic acid and 4-hydroxy benzoic acid also acted as strong anti-lipase inhibitors, with IC_50_ values of 0.08 and 0.16 mg/mL, respectively [44]. Gallic acid also acted as a competitive inhibitor against α-glucosidase, with IC_50_ value of 220.12 µg/mL, which was stronger than acarbose, an anti-diabetic drug with IC_50_ value of 823 µg/mL [45]. Gallic acid in combination with acarbose (3:1 ratio) also effectively inhibited α-amylase (82.2% inhibition), which was stronger than gallic acid (49% inhibition with 25 µM) but similar to acarbose (82.8% inhibition with 25 µM) [46]. These results suggested the reduction in usage of the synthetic drug with synergistic effect with natural bioactive compound.

Interestingly, all fruit extracts exhibited anti-mutagenicity against three mutagens found in overcooked meat including 3-amino-1,4 dimethyl-5H-pyrido [4,3 b]indole (Trp-P1), 3-amino-1-methyl-5H-pyrido[4.3-b]indole (Trp-P2) and 2-amino-3,4-dimethylimidazo[4,5-ƒ]quinoline (MeIQ) in a dose-dependent manner assayed by TA98 strain. Overall, *M. indica* ‘Namdokmai’ provided the highest potency of anti-mutagenicity compared to the other extracts. Juice of *M. indica* ‘Alphonso’ grown in India strongly inhibited ethyl methanesulfonate (EMS)-induced mutagenesis in *Escherichia coli*, whereas *M. indica* ‘Badami’ exhibited low anti-mutagenicity against EMS [47], indicating that cultivar type affects the antimutagenic properties of fruits. This trait was also observed in durian, *D. zibethinus* ‘Chanee’ and *D. zibethinus* ‘Monthong’, which exhibited different anti-mutagenicity against MeIQ mutagen. Phytochemicals play a role in the antimutagenic properties of fruits and vegetables. Our results indicated that *M. indica* ‘Namdokmai’ was rich in phenolic acids, 4-hydroxybenzoic acid, and its derivative (gallic acid). Gallic acid inhibited nifuroxazide and aflatoxin B_1_-mediated mutation in *E. coli* and protected DNA from hydrogen peroxide (H_2_O_2_) determined by comet assay [48]. Gallic acid at 120, 250 and 500 µg effectively inhibited 3-(5-nitro-2-furyl)acrylic acid (5NFAA)-induced mutagenicity in *Salmonella typhimurium* TA100 [31]. In addition, gallic acid was more potent than vanillic acid, syringic acid and ferulic acid [31]. According to high-performance liquid chromatography (HPLC) during the Ames test, our *M. indica* ‘Namdokmai’ extract at 200 mg contained gallic acid at 240 µg, which possibly contributed to the antimutagenicity against all tested mutagens observed in Figure 1. Several studies showed that phytochemicals inhibited DNA mutations in various ways, including inhibition of mutagen activation, mutagen blocking and scavenging mutagens [49]. Gallic acid has been shown to have antimutagenic properties via scavenging electrophilic mutagens and blocking a mutagen that was transferred into cytosol [50]. Although there are no direct reports on the effects of both 4-hydroxybenzoic acid and gallic acid on antimutagenic properties against Trp-P1, Trp-P2 and MeIQ, gallic acid contributed to the anti-mutagenicity properties observed in *M. indica* ‘Namdokmai’. Gallic acid, ferulic acid and caffeic acid were also reported for their anti-mutagenicity against sodium azide and 5NFAA in TA100 strain [31]; thus, it was not surprising that both pineapple, *A. comosus* ‘Pattavia’ and *A. comosus* ‘Phulae’, with high ferulic acid and caffeic acid contents were capable of inhibiting Trp-P1, Trp-P2 and MeIQ-induced DNA mutations.

Carcinogenesis is a multi-step physiological process equipped with initiation, promotion and progression [51]. This study revealed that four out of eight fruit extracts including *P. guajava* ‘Kimju’, *P. guajava* ‘Keenok’, *C. papaya* ‘Khaekdum’ and *M. indica* ‘Namdokmai’ possessed chemopreventive effects against TPA-mediated Raji deformation. *M. indica* ‘Namdokmai’ was determined as the most potent extract. A tumor-promoting agent, 12-O-tetradecanoyl-phorbol-13-acetate (TPA) activated the mitogen-activated protein kinases pathway (MAPK) via phosphorylation of extracellular signal-regulated protein kinase 1 and 2 (ERK1/2) and p38 [52], which leads to Epstein Barr virus (EBV) expression and eventually cell deformation. Thus, one hypothesis postulated from a previous finding suggested that phenolics might contribute to inhibition of Raji deformation. For example, a polyphenol, resveratrol (3,4′,5-trihydroxy-*trans*-stilbene), suppressed the phosphorylation of ERK1/2 and nuclear factor kappa-light-chain-enhancer of activated B cells (NF-kB) pathway when Raji cells were exposed to TPA [52]. As previously stated, *M. indica* ‘Namdokmai’ was rich in 4-hydroxybenzoic acid and gallic acid. It was previously found that gallic acid isolated from *Peltophorum pterocarpum* effectively inhibited deformation of Raji cells [53]. Moreover, gallic acid in various studies showed the same trend on MAPK inhibition; for example, gallic acid at 25–75 µM suppressed ERK1/2 phosphorylation in osteosarcoma cells [54], and MAPK involving proteins ERK1/2 and c-Jun NH_2_-terminal kinase 1 and 2 (JNK1/2) showed reduced phosphorylation after treatment with gallic acid at 30–60 µM in oral cancer cells [55]. In our study, 200 µg/mL of *M. indica* ‘Namdokmai’ contained 1.41 µM of gallic acid, indicating that gallic acid might not solely contribute to the chemopreventive properties observed in Figure 3 and Figure 4; however, the synergistic effects between phenolics and flavonoids must not be neglected [56]. Among the four fruit extracts with anti-deformation effect, three including *P. guajava* ‘Kimju’, *P. guajava* ‘Keenok’ and *M. indica* ‘Namdokmai’ possessed high antioxidant activities. TPA is well-characterized as a tumor-promoting agent but also acts as both an oxidative stress and inflammation inducer [57]. Therefore, these three extracts might exert their chemopreventive effects by quenching TPA-induced oxidative stress. It remains unclear how *C. papaya* ‘Khaekdum’ exhibits chemopreventive properties via the anti-deformation method because the phenolic and flavonoid contents were moderately low. Further determinations on more phytochemical profiles are required to clarify this issue.

In conclusion, other than being nutritionally rich, data from our study both enhance and promote fruit consumption and functional food development. *M. indica* ‘Namdokmai’, a famous cultivar of mango in Thailand, is a rich source of phytochemicals, antioxidants, and NCDs-relevant key enzyme inhibitors, in which consumption of this fruit could possibly reduce DNA mutations. Furthermore, our results showed that different cultivars effectively contributed to phytonutrients and biological activities.

## Figures and Tables

**Table 1:** Phenolic profiles, total phenolic contents (TPCs), and total flavonoid contents (TFCs) of the fruit extracts.

Phenolics (mg/100 g EX)	Fruit Extracts							
	*Psidium guajava*		*Ananas comosus*		*Durio zibethinus*		*Carica papaya*	*Mangifera indica*
	‘Kimju’	‘Keenok’	‘Pattavia’	‘Phulae’	‘Chanee’	‘Monthong’	‘Khaekdum’	‘Namdokmai’
**Phenolic acids**								
Ga	15.53 ± 0.40 ^a,B^	6.32 ± 0.19 ^b,D^	ND	ND	12.83 ± 0.18 ^b,C^	4.35 ± 0.23 ^b,E^	0.51 ± 0.05 ^f,F^	122.05 ± 2.31 ^b,A^
Hy	ND	ND	41.12 ± 1.13 ^b,B^	2.10 ± 0.09 ^f,C^	ND	ND	ND	134.67 ± 2.58 ^a,A^
Va	5.30 ± 0.17 ^d,B^	ND	ND	ND	ND	18.94 ± 0.11 ^a,A^	1.12 ± 0.09 ^e,C^	ND
Sy	4.81 ± 0.22 ^e,A^	ND	ND	ND	ND	0.77 ± 0.01 ^d,B^	ND	ND
Ch	5.51 ± 0.09 ^d,A^	ND	ND	ND	ND	ND	3.31 ± 0.09 ^c,B^	ND
Ca	ND	ND	23.43 ± 0.89 ^c,B^	75.93 ± 1.88 ^a,A^	ND	ND	1.19 ± 0.04 ^e,C^	0.82 ± 0.06 ^e,D^
Fe	ND	ND	112.96 ± 11.36 ^a,A^	26.58 ± 0.45 ^c,B^	1.39 ± 0.06 ^c,A^	ND	1.37 ± 0.03 ^d,C^	ND
Si	ND	ND	ND	ND	ND	ND	11.00 ± 0.25 ^a^	ND
Co	ND	ND	17.03 ± 0.57 ^d,B^	3.92 ± 0.10 ^e,C^	41.85 ± 1.57 ^a,A^	2.19 ± 0.03 ^c,D^	ND	ND
**Flavonoids**								
Na	1.01 ± 0.03 ^f,C^	ND	ND	8.04 ± 0.17 ^d,A^	ND	ND	ND	6.25 ± 0.16 ^d,B^
Ap	ND	ND	ND	2.15 ± 0.05 ^f,A^	ND	ND	0.24 ± 0.02 ^g,B^	ND
Lu	6.15 ± 0.07 ^c,B^	ND	9.44 ± 0.16 ^e,A^	3.45 ± 0.04 ^e,C^	ND	ND	ND	ND
He	ND	ND	ND	52.02 ± 1.31 ^b,A^	ND	ND	5.43 ± 0.05 ^b,C^	9.13 ± 0.26 ^c,B^
Qu	11.52 ± 0.07 ^b,A^	8.94 ± 0.23 ^a,B^	2.07 ± 0.03 ^f,C^	0.47 ± 0.01 ^g,D^	ND	ND	ND	0.32 ± 0.00 ^e,D^
Ka	ND	ND	ND	0.15 ± 0.00 ^g,A^	ND	ND	ND	ND
Is	0.72 ± 0.01 ^g,B^	1.13 ± 0.06 ^c,A^	ND	ND	ND	ND	ND	ND
My	ND	ND	1.01 ± 0.00 ^f,A^	0.94 ± 0.03 ^f,g,B^	ND	ND	ND	ND
**TPCs** **(mg GAE/g EX)**	35.04 ± 1.03 ^C^	28.51 ± 1.40 ^D^	37.28 ± 2.01 ^B^	11.77 ± 0.64 ^E^	8.38 ± 0.17 ^F^	6.60 ± 0.25 ^G^	2.67 ± 0.00 ^H^	94.75 ± 0.35 ^A^
**TFCs** **(mg QE/g EX)**	202.02 ± 12.43 ^B^	79.26 ± 3.84 ^C^	43.05 ± 3.18 ^D^	8.43 ± 0.57 ^E^	286.82 ± 29.47 ^A^	9.99 ± 0.37 ^E^	14.16 ± 1.39 ^E^	16.02 ± 1.08 ^E^

All data are expressed as mean ± standard deviation (SD) of triplicate experiments (*n* = 3). Lower case letters indicate significantly different contents of diverse phenolics detected in the same fruit extracts, while capital letters indicate significantly different contents of the same phenolics detected in diverse fruit extracts at *p* < 0.05 calculated by one–way analysis of variance (ANOVA) and Duncan’s multiple comparison test. EX: extract; ND: not detected; Ga: gallic acid; Hy: 4- hydroxybenzoic acid; Va: vanillic acid; Sy: syringic acid; Ch: chlorogenic acid; Ca: caffeic acid; Fe: ferulic acid; Si: sinapic acid; Co: *p*-coumaric acid; Na: naringenin; Ap: apigenin; Lu: luteolin; He: hesperidin; Qu: quercetin; Ka: kaempferol; Is: isorhamnetin; My: myricetin.

**Table 2:** Antioxidant activities of the fruit extracts.

Fruit Extracts		Antioxidant Activities (μmol TE/g Extract)		
		DPPH Radical Scavenging Assay	FRAP Assay	ORAC Assay
*Psidium guajava*	‘Kimju’	0.40 ± 0.02 ^b^	405.29 ± 8.50 ^b^	1172.90 ± 137.45 ^a^
	‘Keenok’	0.36 ± 0.01 ^c^	379.64 ± 10.19 ^c^	1147.89 ± 101.30 ^a^
*Ananas comosus*	‘Pattavia’	0.16 ± 0.01 ^d^	332.55 ± 12.18 ^d^	1104.50 ± 88.95 ^a^
	‘Phulae’	0.05 ± 0.00 ^e^	163.29 ± 4.13 ^e^	479.76 ± 35.84 ^b^
*Durio zibethinus*	‘Chanee’	0.05 ± 0.00 ^e^	160.88 ± 3.84 ^e^	382.47 ± 11.13 ^b,c^
	‘Monthong’	0.04 ± 0.00 ^e^	77.40 ± 1.02 ^f^	259.55 ± 27.33 ^c^
*Carica papaya*	‘Khaekdum’	0.04 ± 0.00 ^e^	76.24 ± 0.53 ^f^	248.37 ± 15.68 ^c^
*Mangifera indica*	‘Namdokmai’	0.95 ± 0.02 ^a^	691.36 ± 12.67 ^a^	1266.82 ± 145.77 ^a^

All data are expressed as mean ± standard deviation (SD) of triplicate experiments (*n* = 3). Different letters indicate significant differences of the same measurements in diverse fruit extracts at *p* < 0.05 calculated by one-way analysis of variance (ANOVA) and Duncan’s multiple comparison test. DPPH: 2,2-diphenyl-1-picrylhydrazyl; FRAP: ferric reducing antioxidant power; ORAC: oxygen radical antioxidant capacity; TE: Trolox equivalent.

**Table 3:** Enzyme inhibitory activities of the fruit extracts.

Figure		Enzyme Inhibitory Activities (%Inhibition)		
		Lipase ^1^	α-Amylase ^2^	α-Glucosidase ^2^
*Psidium guajava*	‘Kimju’	ND	8.91 ± 0.75 ^c,d^	35.95 ± 0.13 ^b^
	‘Keenok’	ND	12.22 ± 1.33 ^b,c^	34.80 ± 0.13 ^b^
*Ananas comosus*	‘Pattavia’	77.12 ± 0.61 ^a^	13.47 ± 0.06 ^b^	ND
	‘Phulae’	32.10 ± 1.56 ^d^	11.81 ± 0.37 ^b,c^	ND
*Durio zibethinus*	‘Chanee’	71.64 ± 7.42 ^b^	13.68 ± 1.32 ^b^	17.34 ± 0.68 ^e^
	‘Monthong’	25.09 ± 2.31 ^e^	5.79 ± 0.41 ^d,e^	20.16 ± 0.53 ^d^
*Carica papaya*	‘Khaekdum’	ND	5.36 ± 0.22 ^e^	31.09 ± 2.54 ^c^
*Mangifera indica*	‘Namdokmai’	64.87 ± 5.00 ^c^	68.00 ± 4.90 ^a^	93.95 ± 0.58 ^a^

All data are expressed as mean ± standard deviation (SD) of triplicate experiments (*n* = 3). Different letters indicate significantly different enzyme inhibitions of diverse fruit extracts performed under the same enzyme assay at *p* < 0.05 calculated by one-way analysis of variance (ANOVA) and Duncan’s multiple comparison test. ND: not detected; ^1^ concentration of fruit extracts = 0.05 mg/mL; ^2^ concentration of fruit extracts = 0.1 mg/mL.

**Table 4:** Revertant colonies induced by fruit extracts using indicator strain TA98 in S9 mix.

Concentration (mg/plate)	Fruit Extracts							
	*Psidium guajava*		*Ananas comosus*		*Durio zibethinus*		*Carica papaya*	*Mangifera indica*
	‘Kimju’	‘Keenok’	‘Pattavia’	‘Phulae’	‘Chanee’	‘Monthong’	‘Khaekdum’	‘Namdokmai’
0	35 ± 1	37 ± 4	34 ± 2	33 ± 5	25 ± 2	27 ± 1	29 ± 1	27 ± 1
100	36 ± 2	44 ± 1	33 ± 1	37 ± 4	30 ± 4	32 ± 3	32 ± 2	31 ± 3
200	40 ± 3	43 ± 5	36 ± 2	38 ± 2	38 ± 1	32 ± 4	30 ± 3	32 ± 4
300	37 ± 3	36 ± 4	34 ± 2	43 ± 4	31 ± 5	40 ± 6	37 ± 3	42 ± 6

All data are expressed as mean ± standard deviation (SD) of triplicate experiments (*n* = 3). 2-Aminofluorene (2-AF, 1 µg/plate) was used as a positive control, and revertant colonies were 1252 ± 112.

## Data Availability

Data is contained within this article and supplementary material.

## Supplementary Materials

https://www.mdpi.com/article/10.3390/foods10112600/s1. Supplementary Table S1. Images of fruit samples including *Psidium guajava* ‘Kimju’, *Psidium guajava* ‘Keenok’, *Ananas comosus* ‘Pattavia’, *Ananas comosus* ‘Phulae’, *Durio zibethinus* ‘Chanee’, *Durio zibethinus* ‘Monthong’, *Carica papaya* ‘Khaekdum’, and *Mangifera indica* ‘Namdokmai’; Supplementary Figure S1. High-performance liquid chromatograms of phenolic standards including (**A**) gallic acid, (**B**) 4-hydroxybenzoic acid, (**C**) vanillic acid, (**D**) syringic acid, (**E**) hesperidin and (**F**) naringenin and samples including (**G**) *Psidium guajava* ‘Kimju’, (**H**) *Psidium guajava* ‘Keenok’, (**I**) *Ananas comosus* ‘Pattavia’, (**J**) *Ananas comosus* ‘Phulae’, (**K**) *Durio zibethinus* ‘Chanee’, (**L**) *Durio zibethinus* ‘Monthong’, (**M**) *Carica papaya* ‘Khaekdum’, and (**N**) *Mangifera indica* ‘Namdokmai’. Retention times (*R_t_*) of phenolics in fruit extracts are indicated at a wavelength of 280 nm; Supplementary Figure S2. High-performance liquid chromatograms of phenolic standards including (**A**) chlorogenic acid, (**B**) caffeic acid, (**C**) *p*-coumaric acid, (**D**) ferulic acid, and (**E**) sinapic acid, and samples including (**F**) *Psidium guajava* ‘Kimju’, (**G**) *Psidium guajava* ‘Keenok’, (**H**) *Ananas comosus* ‘Pattavia’, (**I**) *Ananas comosus* ‘Phulae’, (**J**) *Durio zibethinus* ‘Chanee’, (**K**) *Durio zibethinus* ‘Monthong’, (**L**) *Carica papaya* ‘Khaekdum’, and (**M**) *Mangifera indica* ‘Namdokmai’. Retention times (*R_t_*) of phenolics in fruit extracts are indicated at a wavelength of 325 nm; Supplementary Figure S3. High-performance liquid chromatograms of phenolic standards including (**A**) luteolin, and (**B**) apigenin, and samples including (**C**) *Psidium guajava* ‘Kimju’, (**D**) *Psidium guajava* ‘Keenok’, (E) *Ananas comosus* ‘Pattavia’, (**F**) *Ananas comosus* ‘Phulae’, (**G**) *Durio zibethinus* ‘Chanee’, (**H**) *Durio zibethinus* ‘Monthong’, (**I**) *Carica papaya* ‘Khaekdum’, and (**J**) *Mangifera indica* ‘Namdokmai’. Retention times (*R_t_*) of phenolics in fruit extracts are indicated at a wavelength of 338 nm; Supplementary Figure S4. High-performance liquid chromatograms of standards including (**A**) myricetin, (**B**) quercetin, (**C**) kaempferol, and (**D**) isorhamnetin and samples including (**E**) *Psidium guajava* ‘Kimju’, (**F**) *Psidium guajava* ‘Keenok’, (**G**) *Ananas comosus* ‘Pattavia’, (**H**) *Ananas comosus* ‘Phulae’, (**I**) *Durio zibethinus* ‘Chanee’, (**J**) *Durio zibethinus* ‘Monthong’, (**K**) *Carica papaya* ‘Khaekdum’, and (**L**) *Mangifera indica* ‘Namdokmai’. Retention times (*R_t_*) of phenolics in fruit extracts are indicated at a wavelength of 368 nm; Supplementary Figure S5. The cell morphology of Raji cells. (**A**) Negative control contained 0.1% (*v*/*v*) dimethyl sulfoxide (DMSO) and 1 mM sodium butyrate, and (**B**) positive control contained 0.1% (*v*/*v*) DMSO, 1 mM sodium butyrate, and 1 mM TPA. In this positive control, cells were clearly deformed into tree branch-like, dilation and flatness. Upper picture magnification: 20X and lower picture magnification: 40X; Supplementary Figure S6. The cell morphology of Raji cells after treatment with 0.1% (*v*/*v*) dimethyl sulfoxide (DMSO), 1 mM sodium butyrate, 1 mM and each fruit extracts from 1–200 µg/mL. Upper picture magnification: 20X and lower picture magnification: 40X. (**A**) *Psidium guajava* ‘Kimju’, (**B**) *Psidium guajava* ‘Keenok’, (**C**) *Ananas comosus* ‘Pattavia’, (**D**) *Durio zibethinus* ‘Chanee’, (**E**) *Durio zibethinus* ‘Monthong’, and (**F**) *Carica papaya* ‘Khaekdum’.
