# Supplementary material for: Analysis of Phytonutrients, Anti-Mutagenic and Chemopreventive Effects of Tropical Fruit Extracts
Source: Foods. 2021 Oct 27;10(11):2600. doi: 10.3390/foods10112600 (PMC8621897; doi:10.3390/foods10112600)

foods

Supplementary materials

Analysis of Phytonutrients, Anti-mutagenic and Chemopreventive Effects of Tropical Fruit Extracts

Piya Temviriyanukul [1], Suwapat Kittibunchakul [1], Piyapat Trisonthi [2], Woorawee Inthachat [1], Dalad Siriwan [2,*,†] and Uthaiwan Suttisansanee [1,*,†]

1 Food and Nutrition Academic and Research Cluster, Institute of Nutrition, Mahidol University, Salaya, Phuttamonthon, Nakhon Pathom 73170, Thailand; piya.tem@mahidol.ac.th (P.T.); suwapat.kit@mahidol.ac.th (S.K.); woorawee.int@mahidol.ac.th (W.I.)
2 Institute of Food Research and Product Development, Kasetsart University, Chatuchak, Bangkok 10900, Thailand; piyapat.tr@ku.th (P.Tr).
* Correspondence: dalad.s@ku.th (D.S.); uthaiwan.sut@mahidol.ac.th (U.S.)
† These authors contributed equally to this work.

Supplementary Table S1:

Images of fruit samples including *Psidium guajava* 'Kimju', *Psidium guajava* 'Keenok', *Ananas comosus* 'Pattavia', *Ananas comosus* 'Phulae', *Durio zibethinus* 'Chanee', *Durio zibethinus* 'Monthong', *Carica papaya* 'Khaekdum', and *Mangifera indica* 'Namdokmai'.

Fruits	Physical appearance	
Psidium guajava	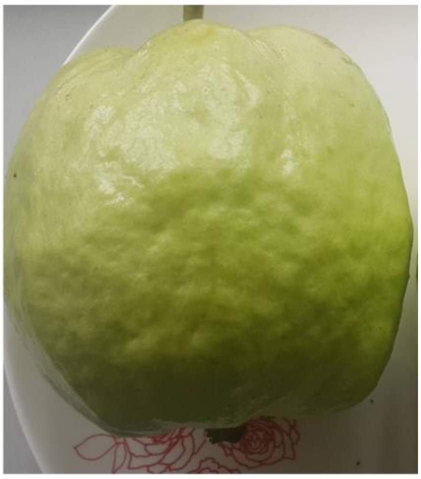 'Kimju'	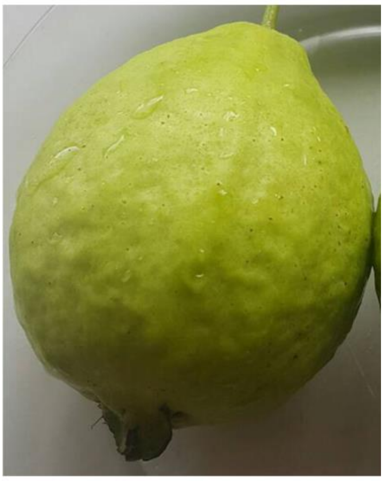 'Keenok'
Ananas comosus	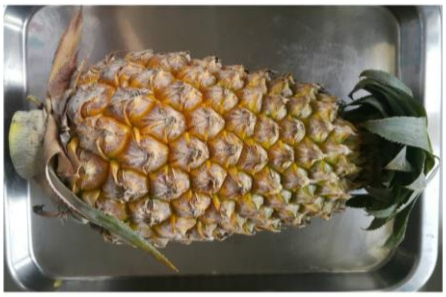 'Pattavia'	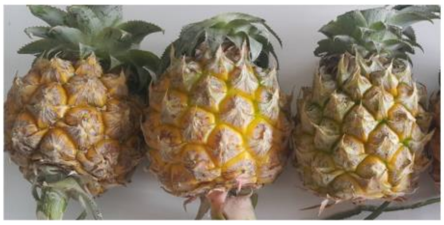 'Phulae'
Durio zibethinus	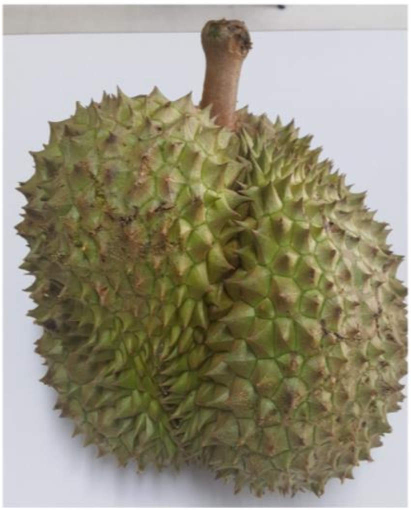 'Chanee'	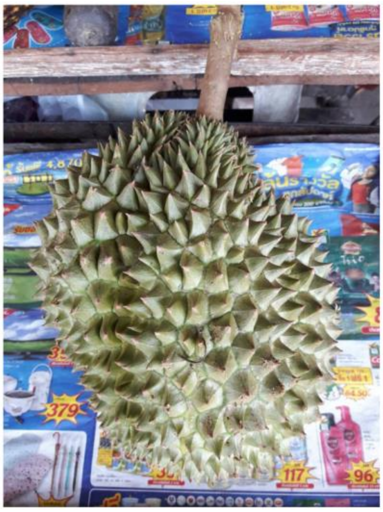 'Monthong'

Supplementary Table S1 (Cont.):

Images of fruit samples including *Psidium guajava* 'Kimju', *Psidium guajava* 'Keenok', *Ananas comosus* 'Pattavia', *Ananas comosus* 'Phulae', *Durio zibethinus* 'Chanee', *Durio zibethinus* 'Monthong', *Carica papaya* 'Khaekdum', and *Mangifera indica* 'Namdokmai'.

Fruits	Physical appearance
Carica papaya 'Khaekdum'	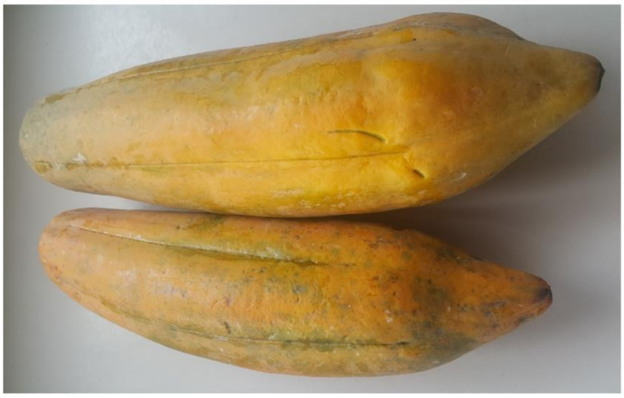
Mangifera indica 'Namdokmai'	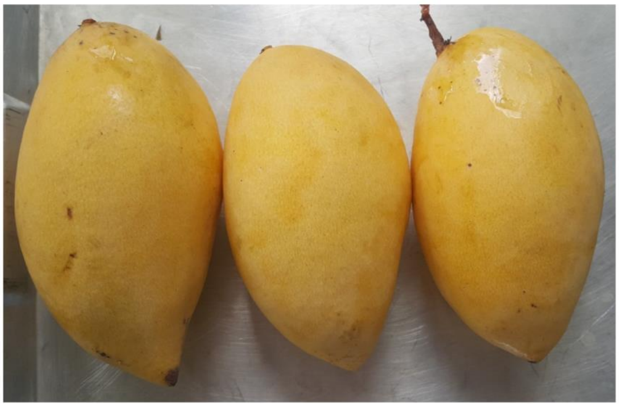

Supplementary Figure S1:

High-performance liquid chromatograms of phenolic standards including (A) gallic acid, (B) 4-hydroxybenzoic acid, (C) vanillic acid, (D) syringic acid, (E) hesperidin and (F) naringenin and samples including (G) *Psidium guajava* 'Kimju', (H) *Psidium guajava* 'Keenok', (I) *Ananas comosus* 'Pattavia', (J) *Ananas comosus* 'Phulae', (K) *Durio zibethinus* 'Chanee', (L) *Durio zibethinus* 'Monthong', (M) *Carica papaya* 'Khaekdum', and (N) *Mangifera indica* 'Namdokmai'. Retention times (R_t) of phenolics in fruit extracts are indicated at a wavelength of 280 nm.

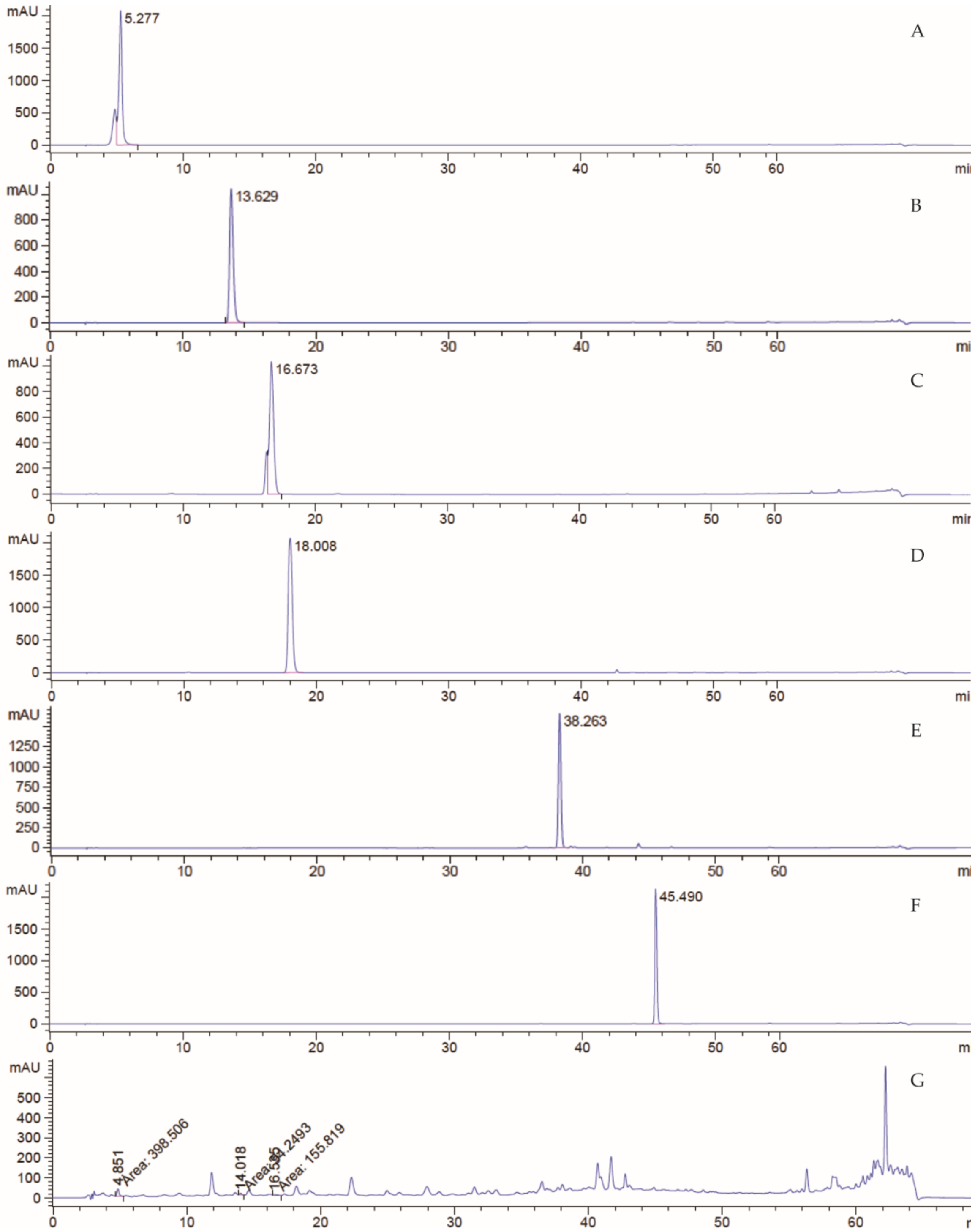

Supplementary Figure S1 (Cont.):

High-performance liquid chromatograms of phenolic standards including (A) gallic acid, (B) 4-hydroxybenzoic acid, (C) vanillic acid, (D) syringic acid, (E) hesperidin and (F) naringenin and samples including (G) *Psidium guajava* 'Kimju', (H) *Psidium guajava* 'Keenok', (I) *Ananas comosus* 'Pattavia', (J) *Ananas comosus* 'Phulae', (K) *Durio zibethinus* 'Chanee', (L) *Durio zibethinus* 'Monthong', (M) *Carica papaya* 'Khaekdum', and (N) *Mangifera indica* 'Namdokmai'. Retention times (R_t) of phenolics in fruit extracts are indicated at a wavelength of 280 nm.

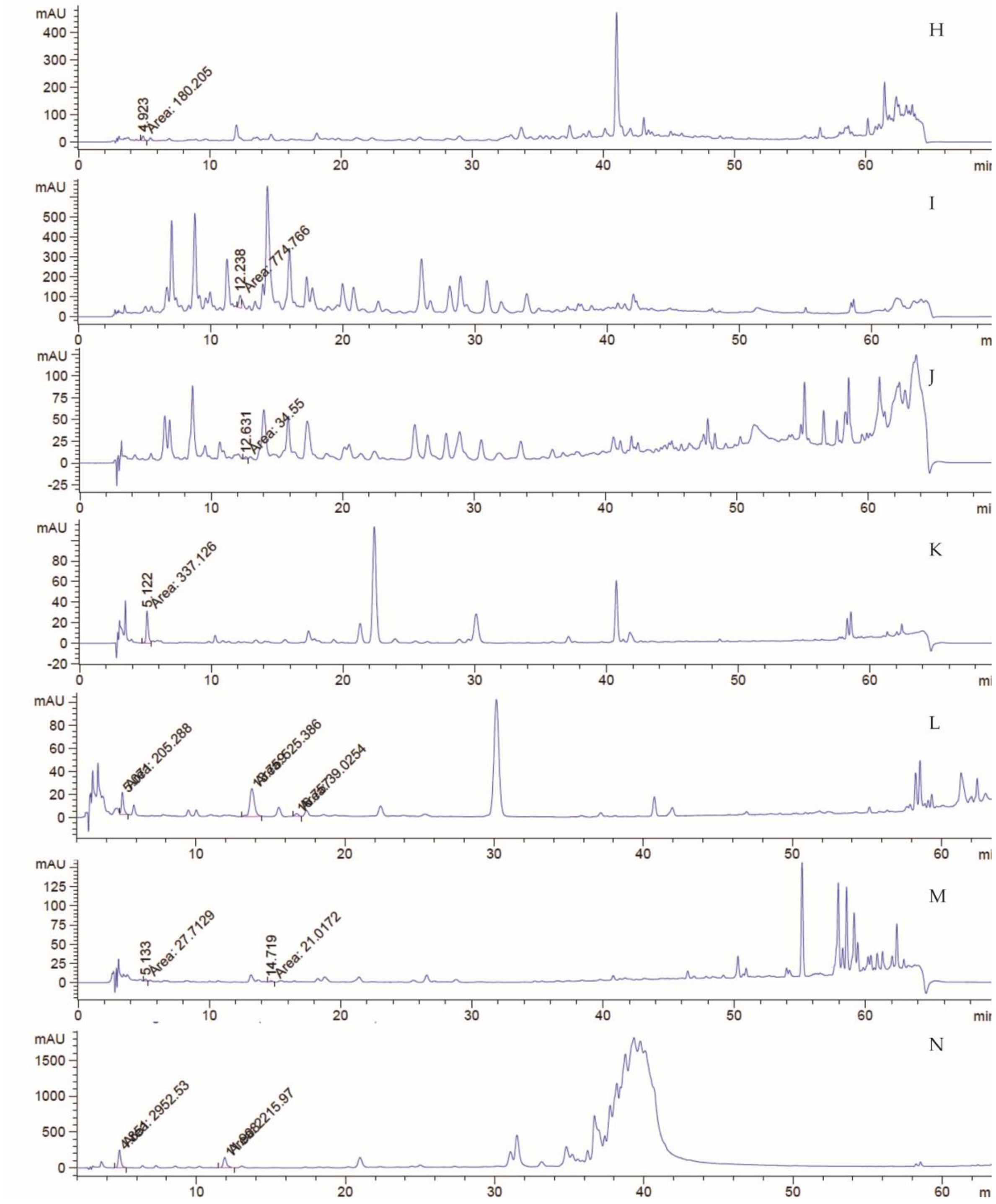

Supplementary Figure S2:

High-performance liquid chromatograms of phenolic standards including (A) chlorogenic acid, (B) caffeic acid, (C) *p*-coumaric acid, (D) ferulic acid, and (E) sinapic acid, and samples including (F) *Psidium guajava* 'Kimju', (G) *Psidium guajava* 'Keenok', (H) *Ananas comosus* 'Pattavia', (I) *Ananas comosus* 'Phulae', (J) *Durio zibethinus* 'Chanee', (K) *Durio zibethinus* 'Monthong', (L) *Carica papaya* 'Khaekdum', and (M) *Mangifera indica* 'Namdokmai'. Retention times (R_t) of phenolics in fruit extracts are indicated at a wavelength of 325 nm.

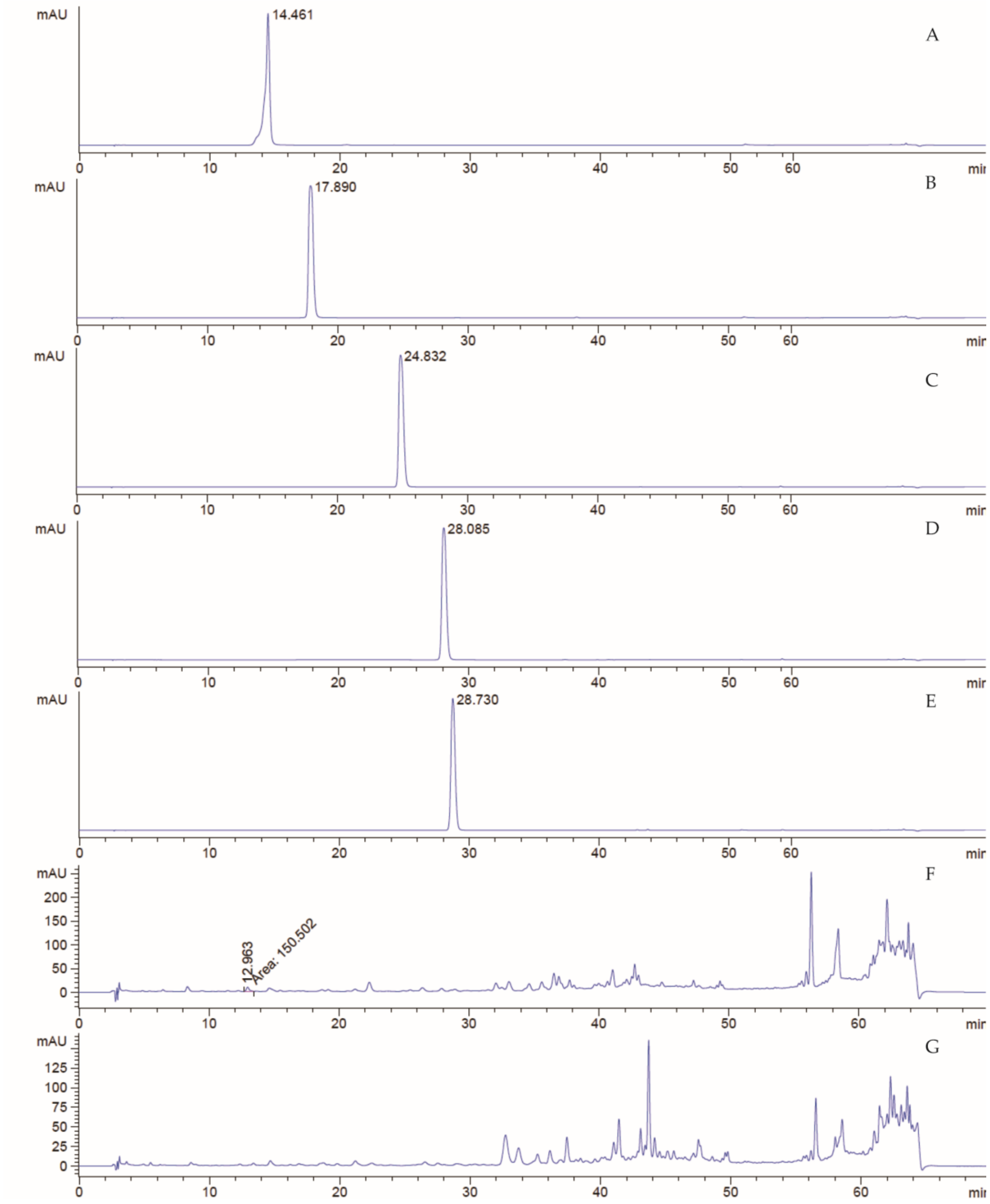

Supplementary Figure S2 (Cont.):

High-performance liquid chromatograms of phenolic standards including (A) chlorogenic acid, (B) caffeic acid, (C) *p*-coumaric acid, (D) ferulic acid, and (E) sinapic acid, and samples including (F) *Psidium guajava* 'Kimju', (G) *Psidium guajava* 'Keenok', (H) *Ananas comosus* 'Pattavia', (I) *Ananas comosus* 'Phulae', (J) *Durio zibethinus* 'Chanee', (K) *Durio zibethinus* 'Monthong', (L) *Carica papaya* 'Khaekdum', and (M) *Mangifera indica* 'Namdokmai'. Retention times (R_t) of phenolics in fruit extracts are indicated at a wavelength of 325 nm.

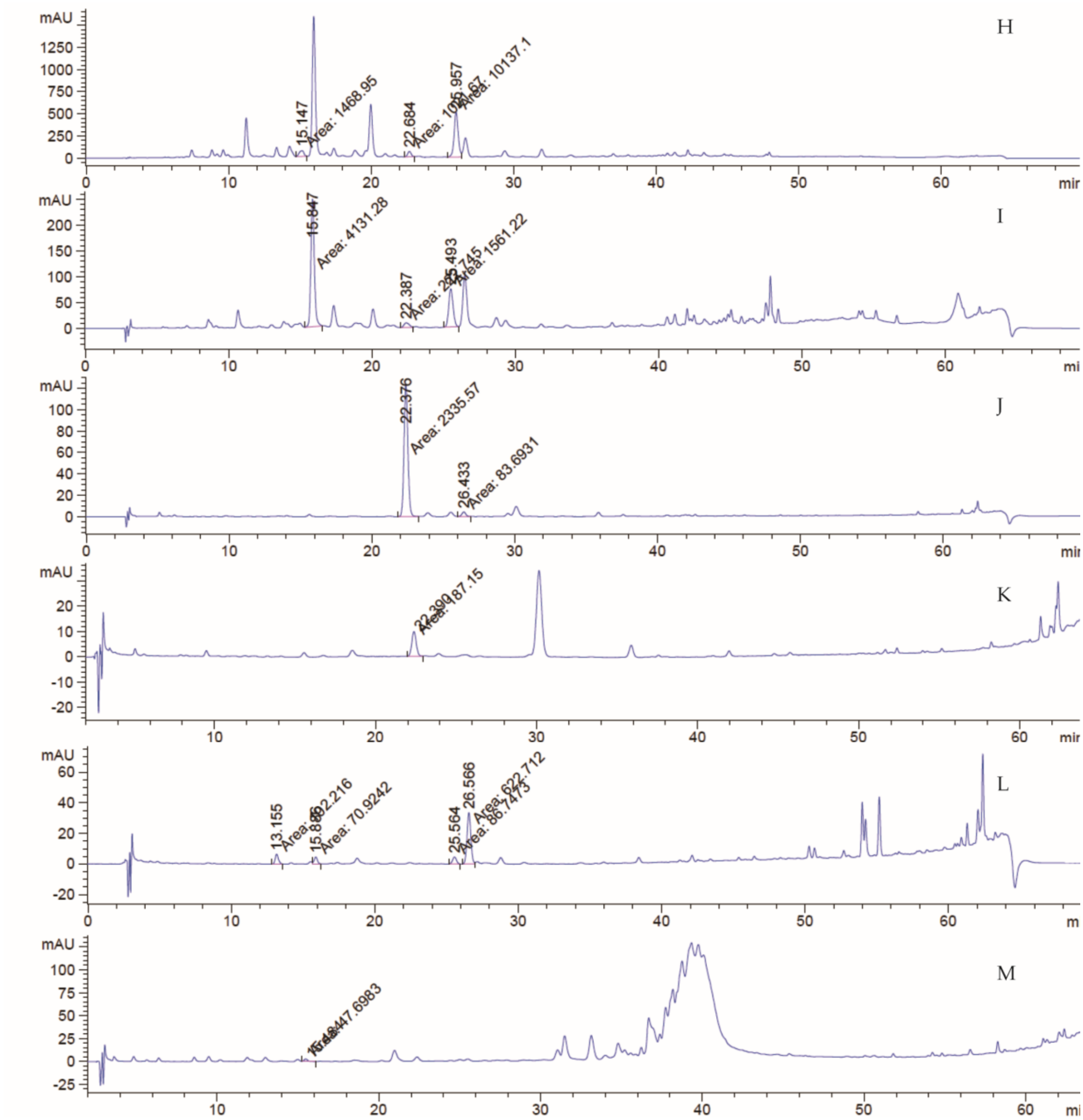

Supplementary Figure S3:

High-performance liquid chromatograms of phenolic standards including (A) luteolin, and (B) apigenin, and samples including (C) *Psidium guajava* 'Kimju', (D) *Psidium guajava* 'Keenok', (E) *Ananas comosus* 'Pattavia', (F) *Ananas comosus* 'Phulae', (G) *Durio zibethinus* 'Chanee', (H) *Durio zibethinus* 'Monthong', (I) *Carica papaya* 'Khaekdum', and (J) *Mangifera indica* 'Namdokmai'. Retention times (R_t) of phenolics in fruit extracts are indicated at a wavelength of 338 nm.

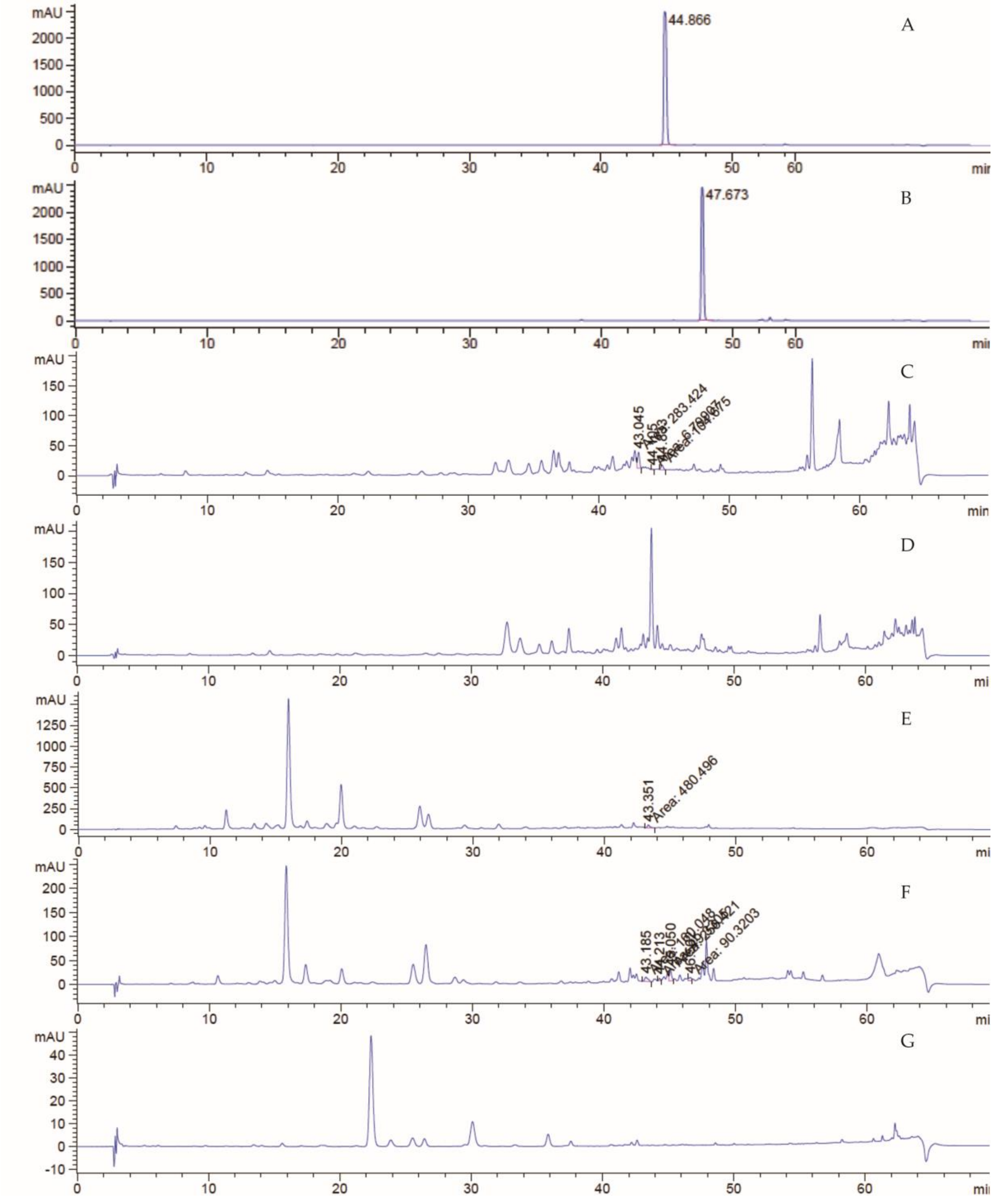

Supplementary Figure S3 (Cont.):

High-performance liquid chromatograms of phenolic standards including (A) luteolin, and (B) apigenin, and samples including (C) *Psidium guajava* 'Kimju', (D) *Psidium guajava* 'Keenok', (E) *Ananas comosus* 'Pattavia', (F) *Ananas comosus* 'Phulae', (G) *Durio zibethinus* 'Chanee', (H) *Durio zibethinus* 'Monthong', (I) *Carica papaya* 'Khaekdum', and (J) *Mangifera indica* 'Namdokmai'. Retention times (R_t) of phenolics in fruit extracts are indicated at a wavelength of 338 nm.

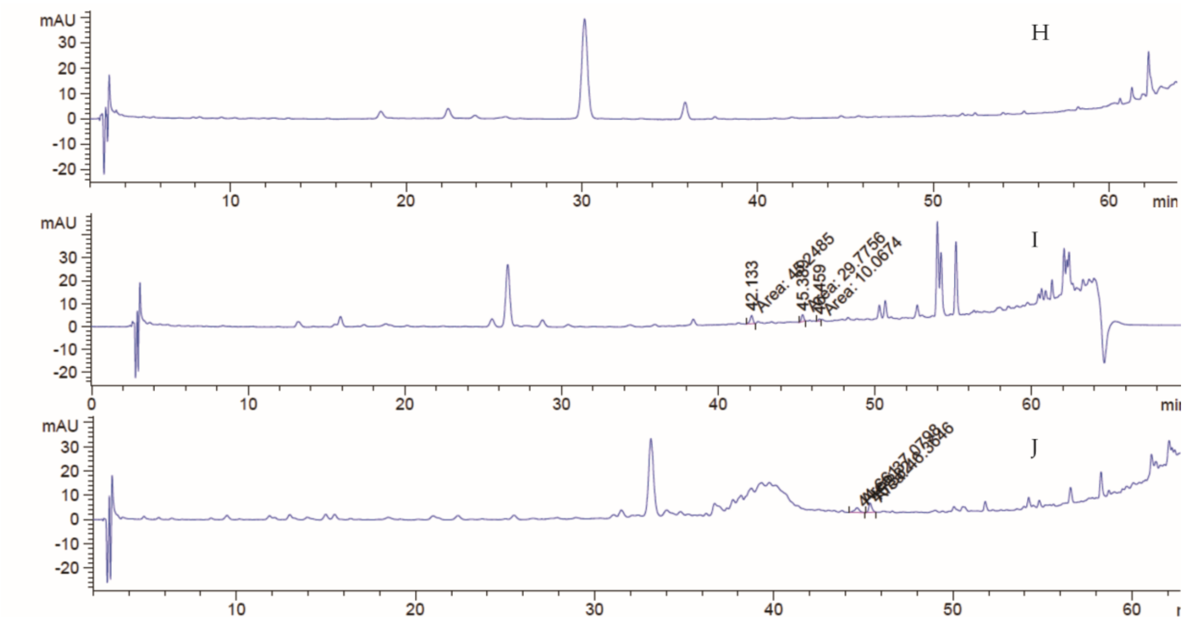

Supplementary Figure S4:

High-performance liquid chromatograms of standards including (A) myricetin, (B) quercetin, (C) kaempferol, and (D) isorhamnetin and samples including (E) *Psidium guajava* 'Kimju', (F) *Psidium guajava* 'Keenok', (G) *Ananas comosus* 'Pattavia', (H) *Ananas comosus* 'Phulae', (I) *Durio zibethinus* 'Chanee', (J) *Durio zibethinus* 'Monthong', (K) *Carica papaya* 'Khaekdum', and (L) *Mangifera indica* 'Namdokmai'. Retention times (R_t) of phenolics in fruit extracts are indicated at a wavelength of 368 nm.

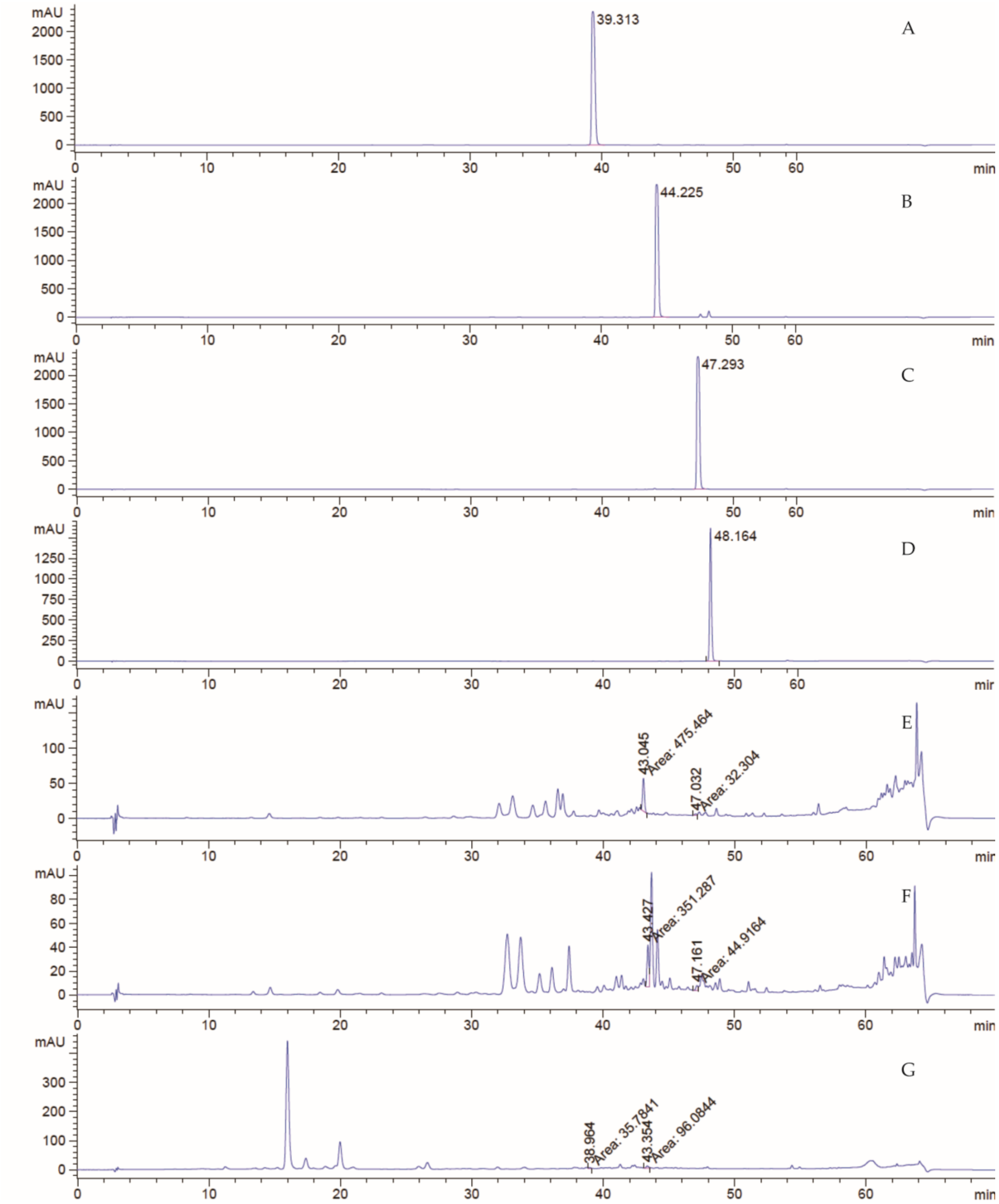

Supplementary Figure S4 (Cont.):

High-performance liquid chromatograms of standards including (A) myricetin, (B) quercetin, (C) kaempferol, and (D) isorhamnetin and samples including (E) *Psidium guajava* 'Kimju', (F) *Psidium guajava* 'Keenok', (G) *Ananas comosus* 'Pattavia', (H) *Ananas comosus* 'Phulae', (I) *Durio zibethinus* 'Chanee', (J) *Durio zibethinus* 'Monthong', (K) *Carica papaya* 'Khaekdum', and (L) *Mangifera indica* 'Namdokmai'. Retention times (R_t) of phenolics in fruit extracts are indicated at a wavelength of 368 nm.

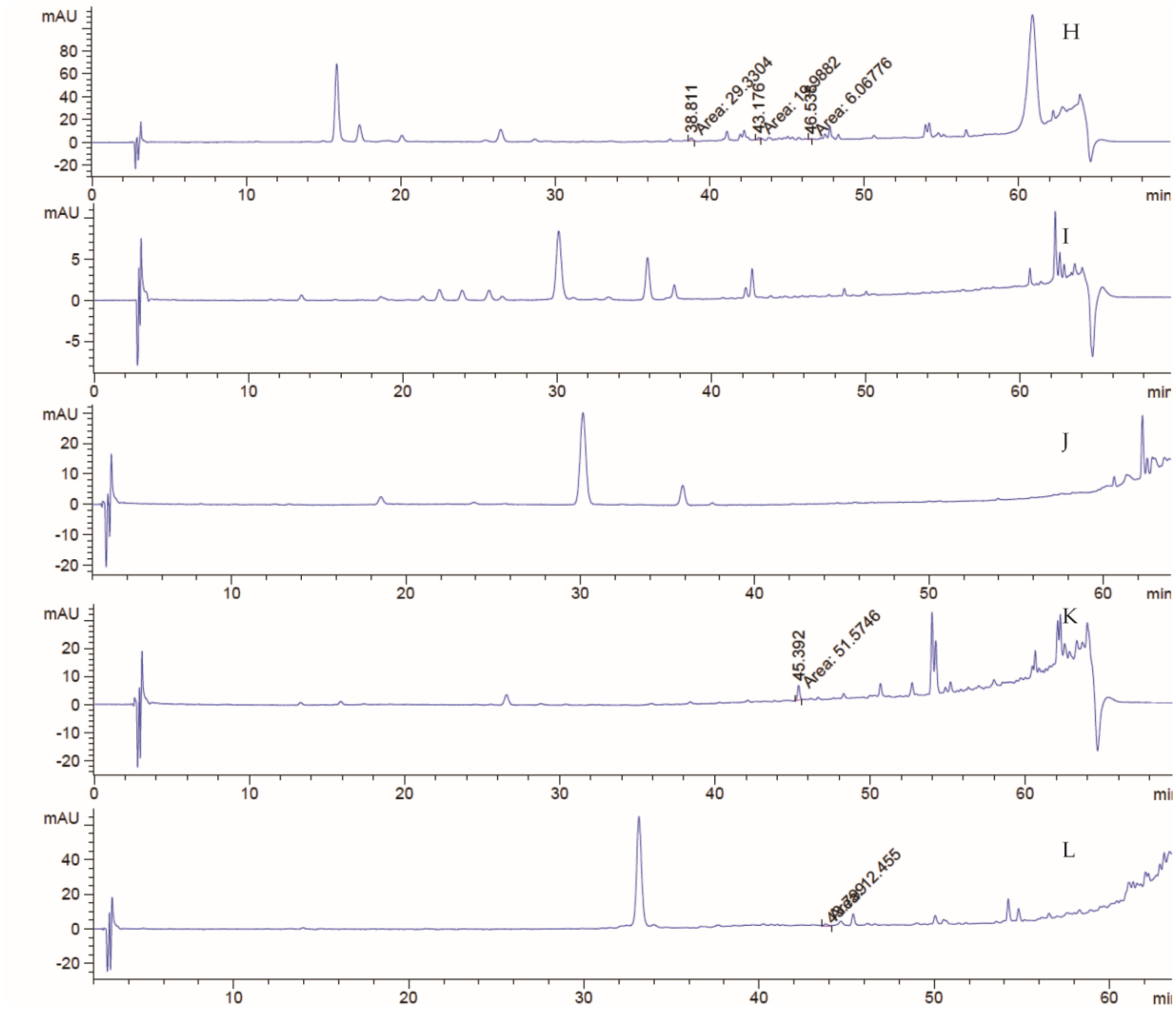

Supplementary Figure S5:

The cell morphology of Raji cells. (A) Negative control contained 0.1% (v/v) dimethyl sulfoxide (DMSO) and 1 mM sodium butyrate, and (B) positive control contained 0.1% (v/v) DMSO, 1 mM sodium butyrate, and 1 mM TPA. In this positive control, cells were clearly deformed into tree branch-like, dilation and flatness. Upper picture magnification: 20X and lower picture magnification: 40X;

A. B.

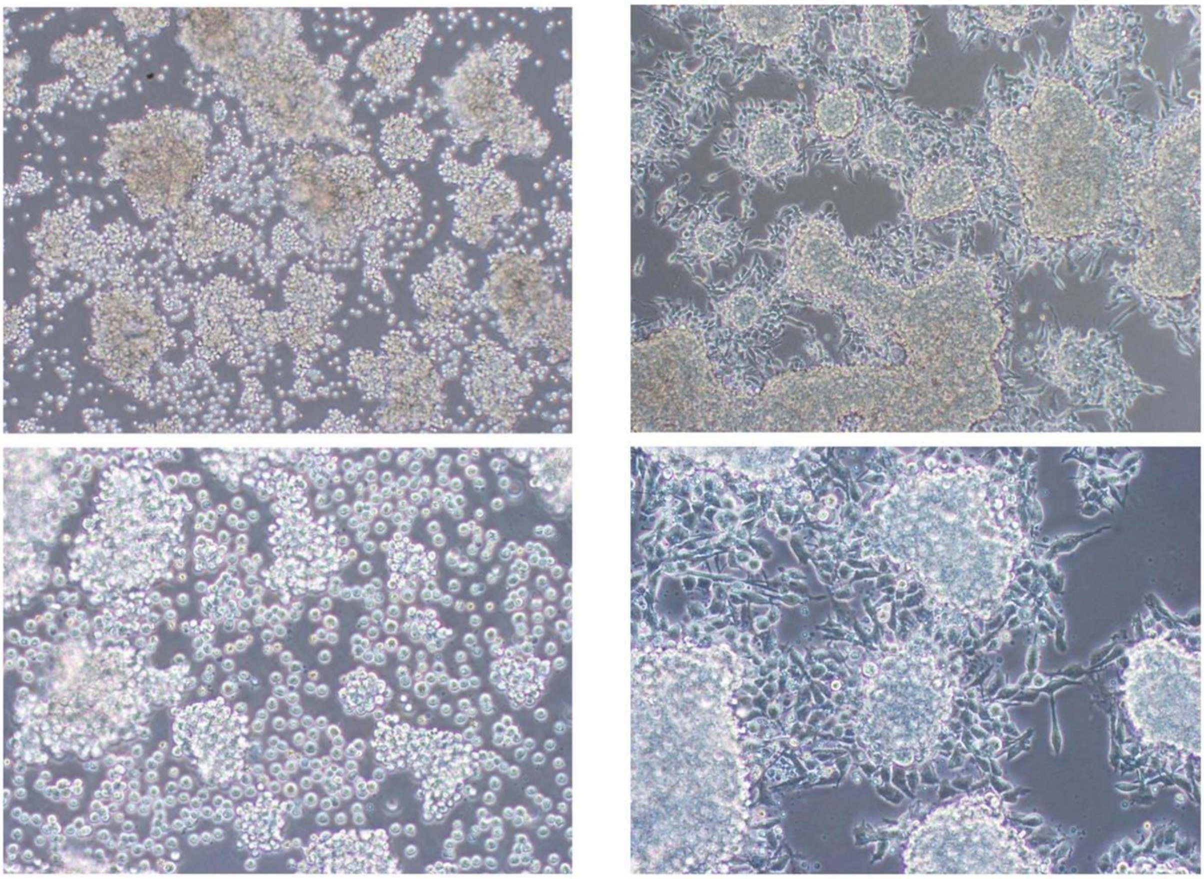

Supplementary Figure S6: The cell morphology of Raji cells after treatment with 0.1% (v/v) dimethyl sulfoxide (DMSO), 1 mM sodium butyrate, 1 mM and each fruit extracts from 1-200 µg/mL. Upper picture magnification: 20X and lower picture magnification: 40X. (A) *Psidium guajava* 'Kimju', (B) *Psidium guajava* 'Keenok', (C) *Ananas comosus* 'Pattavia',

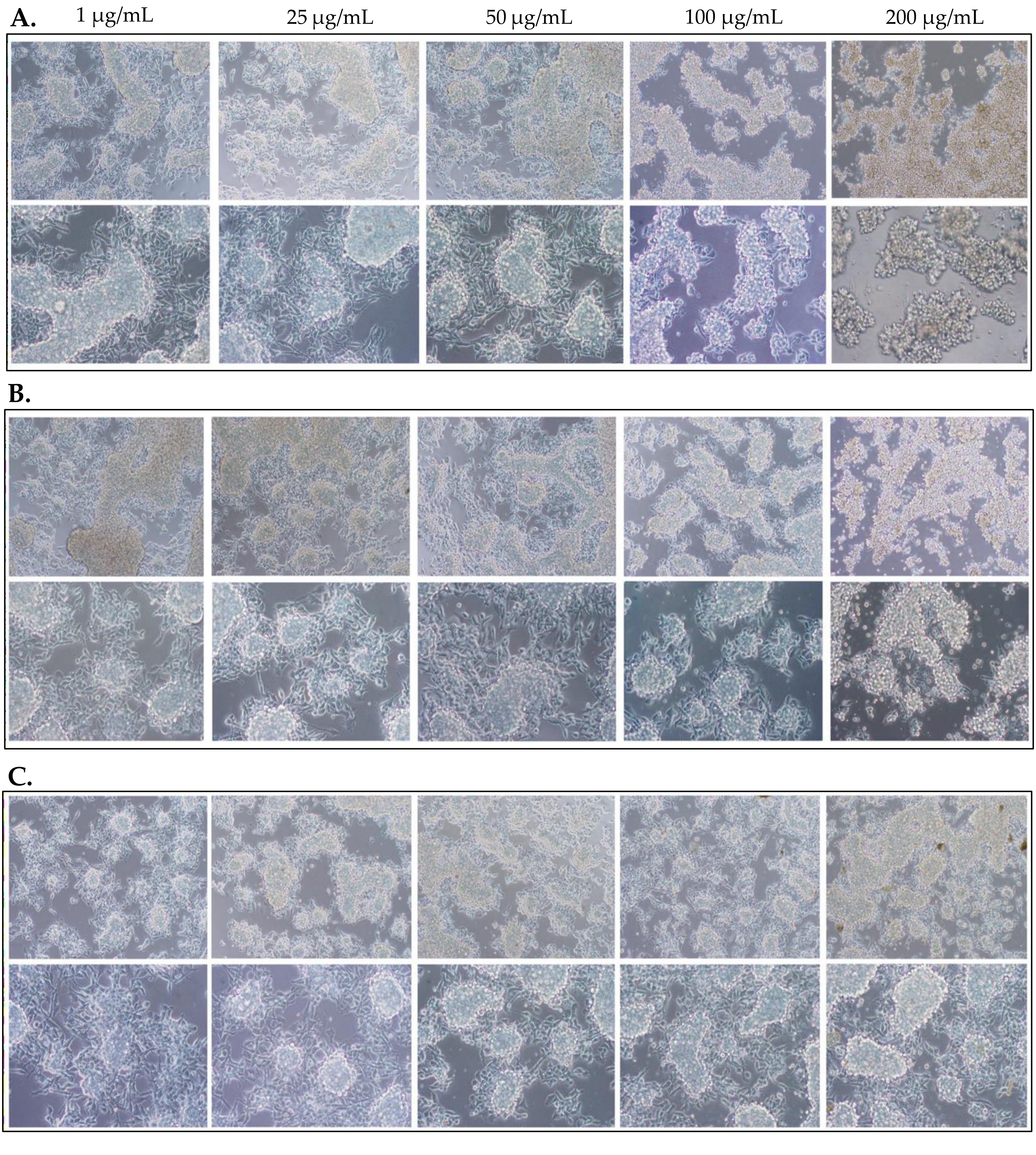

Supplementary Figure S6 (Cont.): The cell morphology of Raji cells after treatment with 0.1% DMSO, 1 mM sodium butyrate , 1 mM and each fruit extracts from 1-200 μg/mL. Upper picture magnification: 20X and lower picture magnification: 40X. (D) *Durio zibethinus* 'Chanee', (E) *Durio zibethinus* 'Monthong', and (F) *Carica papaya* 'Khaekdum'.

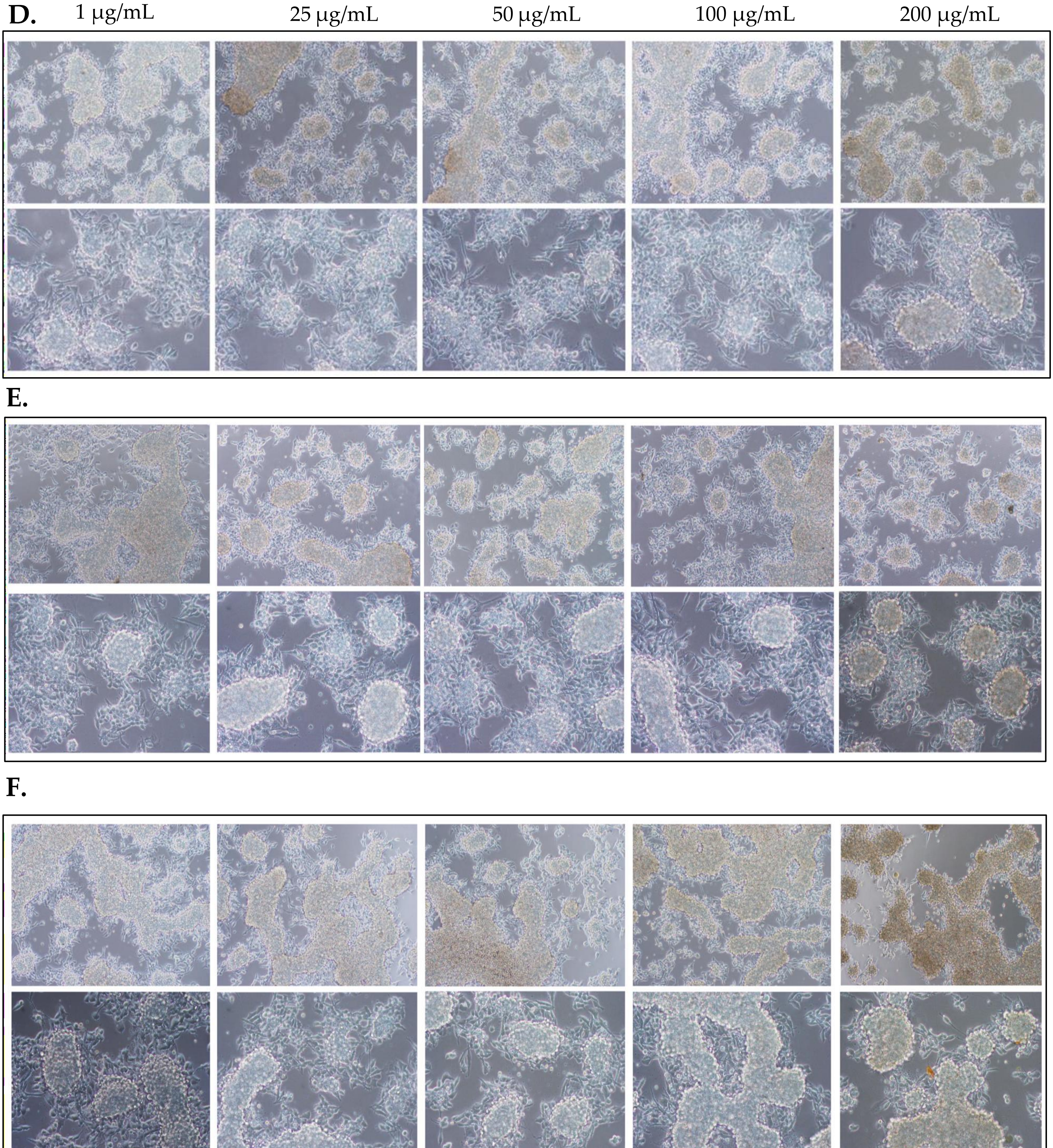

Supplement: Supplementary file 1 [file foods-10-02600-s001.zip › foods-1400728-SI.pdf]
